# Comprehensive probiogenomics analysis of the commensal *Escherichia coli* CEC15 as a potential probiotic strain

**DOI:** 10.1186/s12866-023-03112-4

**Published:** 2023-11-27

**Authors:** Tales Fernando da Silva, Rafael de Assis Glória, Thiago Jesus de Sousa, Monique Ferrary Americo, Andria dos Santos Freitas, Marcus Vinicius Canário Viana, Luís Cláudio Lima de Jesus, Ligia Carolina da Silva Prado, Nathalie Daniel, Olivia Ménard, Marie-Françoise Cochet, Didier Dupont, Julien Jardin, Amanda Dias Borges, Simone Odília Antunes Fernandes, Valbert Nascimento Cardoso, Bertram Brenig, Enio Ferreira, Rodrigo Profeta, Flavia Figueira Aburjaile, Rodrigo Dias Oliveira de Carvalho, Philippe Langella, Yves Le Loir, Claire Cherbuy, Gwénaël Jan, Vasco Azevedo, Éric Guédon

**Affiliations:** 1 1INRAE, Institut Agro, STLO, UMR1253, 65 rue de Saint Brieuc, 35042 Rennes, Cedex France; 2 Department of Genetics, Ecology, and Evolution, Institute of Biological Sciences, Federal University of Minas Gerais, Belo Horizonte, Brazil; 3 Department of clinical and toxicological analysis, Faculty of Pharmacy, Federal University of Minas Gerais, Belo Horizonte, Brazil; 4 Department of Molecular Biology of Livestock, Institute of Veterinary Medicine, Georg-August Universität Göttingen, Göttingen, Germany; 5 Department of general pathology, Federal University of Minas Gerais, Belo Horizonte, Brazil; 6 Veterinary school, Federal University of Minas Gerais, Belo Horizonte, Brazil; 7 Federal University of Bahia, Salvador, Brazil; 8 Université Paris Saclay, INRAE, AgroParisTech, UMR1319, MICALIS, Jouy-en-Josas, France

**Keywords:** Probiotics, Probiogenomics, *Escherichia coli* CEC15, Genomics, *Escherichia coli* Nissle 1917, Mucositis, Immunomodulation, Gastrointestinal tract

## Abstract

**Background:**

Probiotics have gained attention for their potential maintaining gut and immune homeostasis. They have been found to confer protection against pathogen colonization, possess immunomodulatory effects, enhance gut barrier functionality, and mitigate inflammation. However, a thorough understanding of the unique mechanisms of effects triggered by individual strains is necessary to optimize their therapeutic efficacy. Probiogenomics, involving high-throughput techniques, can help identify uncharacterized strains and aid in the rational selection of new probiotics. This study evaluates the potential of the *Escherichia coli* CEC15 strain as a probiotic through *in silico*, *in vitro*, and *in vivo* analyses, comparing it to the well-known probiotic reference *E. coli* Nissle 1917. Genomic analysis was conducted to identify traits with potential beneficial activity and to assess the safety of each strain (genomic islands, bacteriocin production, antibiotic resistance, production of proteins involved in host homeostasis, and proteins with adhesive properties). *In vitro* studies assessed survival in gastrointestinal simulated conditions and adhesion to cultured human intestinal cells. Safety was evaluated in BALB/c mice, monitoring the impact of *E. coli* consumption on clinical signs, intestinal architecture, intestinal permeability, and fecal microbiota. Additionally, the protective effects of both strains were assessed in a murine model of 5-FU-induced mucositis.

**Results:**

CEC15 mitigates inflammation, reinforces intestinal barrier, and modulates intestinal microbiota. *In silico* analysis revealed fewer pathogenicity-related traits in CEC15, when compared to Nissle 1917, with fewer toxin-associated genes and no gene suggesting the production of colibactin (a genotoxic agent). Most predicted antibiotic-resistance genes were neither associated with actual resistance, nor with transposable elements. The genome of CEC15 strain encodes proteins related to stress tolerance and to adhesion, in line with its better survival during digestion and higher adhesion to intestinal cells, when compared to Nissle 1917. Moreover, CEC15 exhibited beneficial effects on mice and their intestinal microbiota, both in healthy animals and against 5FU-induced intestinal mucositis.

**Conclusions:**

These findings suggest that the CEC15 strain holds promise as a probiotic, as it could modulate the intestinal microbiota, providing immunomodulatory and anti-inflammatory effects, and reinforcing the intestinal barrier. These findings may have implications for the treatment of gastrointestinal disorders, particularly some forms of diarrhea.

**Supplementary Information:**

The online version contains supplementary material available at 10.1186/s12866-023-03112-4.

## Background

Probiotics are commonly used to mitigate the severity of certain illnesses, such as diarrhea caused by antibiotics, childhood diarrhea, ulcerative colitis, pouchitis, and eczema associated with cow's milk allergy [1]. Probiotics are “live microorganisms that when administered in adequate amounts confer health benefits on the host” [2], and it is important to note that each probiotic strain has specific effects, and the success of one strain does not guarantee the success of another. The genetic differences between probiotic bacteria can be greater than the differences between humans and goldfish [3]. While some characteristics, like safety status, are common among probiotic species, mechanisms for probiotic activity are less common and only present in certain strains (strain-dependent effect) [4–6]. For example, strains of *Enterococcus faecium* can be beneficial as a probiotic, while other strains of the same species can also be pathogens that cause problems due to antibiotic resistance [1]. The most common probiotics belongs to the lactic acid bacteria (LAB) group, the genera *Bifidobacterium* and *Propionibacterium*, and the yeast *Saccharomyces* [7]. There is one Gram-negative bacterium, which has been considered as a probiotic due to its protective effect against enteropathogenic bacteria, the *Escherichia coli* Nissle 1917 strain [8].

The *E. coli* Nissle 1917 strain (hereafter referred to as EcN) was first isolated in 1915 from feces of a soldier by German army physician Alfred Nissle [9, 10]. This strain presented good antagonistic effects against the bacteria that were causing a diarrhea outbreak at the moment, i.e. *Salmonella enterica* serovar Paratyphi, *Shigella dysenteriae*, *S*. *flexnery*, *Proteus vulgaris* and *P*. *mirabilis* [10]. A preparation containing EcN (Mutaflor®) was administered to the sick soldiers and was able to restore the healthy state on them [11]. Over a century later, this strain is still being used worldwide to treat intestinal infectious diseases [10, 12, 13] and its probiotic activities have been the subject of intensive research [14–20]. However, complete genome sequencing of EcN [21, 22] , as well as the advance of the genomic era evidenced that this strain has genes responsible to produce colibactin, a genotoxic secondary metabolite produced by some enterobacteria, that creates interstrand crosslinks in DNA, which could lead to the development and the progression of colorectal cancer [23, 24]. Furthermore, the beneficial effect of this strain is linked to the presence of colibactin in a way that the knock-out of genes in the referred cluster inhibits greatly the anti-inflammatory effect of the strain on a DSS-induced colitis rat’s model [25, 26]. This has rose concerns regarding the safe use of this strain.

The general ways in which probiotic microorganisms improve human health can be grouped into several categories, such as enhancing the intestinal barrier [27], regulating the immune system [28], and combating harmful pathogens through antimicrobial production [29] or competition for binding sites in the mucus barrier [30]. Although there is some supporting evidence for these claims, the specific molecular processes responsible for these activities are still largely unknown [31].

To select new probiotic strains, microbial cultures from unconventional ecosystems need to undergo a thorough evaluation process, including *in vitro* experiments, animal models, and clinical trials [32]. However, the traditional tests are not always reliable indicators of probiotic safety and efficacy, making it difficult to predict their functionality. Additionally, there are no specific attributes that are essential to all probiotics, and probiotics may exert more than one mechanism associated with a given clinical benefit [33]. These knowledge gaps complicate the efforts to understand and predict the safety and functionality of probiotics. To address these issues, the concept of "probiogenomics" has emerged as a growing area of research interest [34]. Probiogenomics involves high-throughput techniques, such as genomics, transcriptomics, proteomics, and metabolomics, which can provide a useful resource for revealing uncharacterized strains and allow for the design of predictive models for the rational selection of new probiotics [34, 35].

A new strain of *E. coli* with beneficial properties was recently isolated from suckling rodents’ feces [36]. The *E. coli* CEC15 has demonstrated barrier reinforcement effect in the colonic epithelium and anti-inflammatory related immunomodulation on germ-free and conventional mice affected by TNBS-induced colitis and in IL10 -/- mice [37]. These effects suggest a promising effect of the CEC15 strain in the treatment of intestinal inflammatory diseases.

The aim of this work was to make a thorough evaluation of the CEC15 strain through *in silico*, *in vitro*, and *in vivo* analyses on its potential as a probiotic strain, comparing it to the well-known probiotic EcN reference strain. Their genomic composition and their potential for immunomodulation, barrier reinforcement, anti-inflammatory effect, and ability to modulate the intestinal microbiota are the focus of this work.

## Results

### General features of the E. coli CEC15 genome

The complete genome of the *E. coli* CEC15 strain consisted of a circular chromosome of length 4,780,804 bp, with a GC content of 50.66%, and a plasmid of length 200,825 bp with a GC content of 50.7%. The genome annotation showed a total of 4,505 CDS for the chromosome, with 4248 predicted as proteins, 152 being hypothetical proteins, 22 corresponding to rRNA, and 83 to tRNA, while the plasmid presented 213 CDS, from which 40 are hypothetical proteins.

The CEC15 genome was compared with that of the probiotic *E. coli* strain Nissle 1917 (EcN). CEC15 has a slightly smaller genome when compared to EcN (5.05 Mb) presenting 220 fewer CDS (4,725 CDS on the EcN chromosome). On the other hand, CEC15 harbors a larger plasmid in size and number of CDS than the EcN plasmids pMUT1 (3,173 bp with 6 CDS) and pMUT2 (5,514 bp with 8 CDS). CEC15 was classified as *E. coli* serotype O180:H14, while EcN has the serotype O6:H1.

**Fig. 1:**
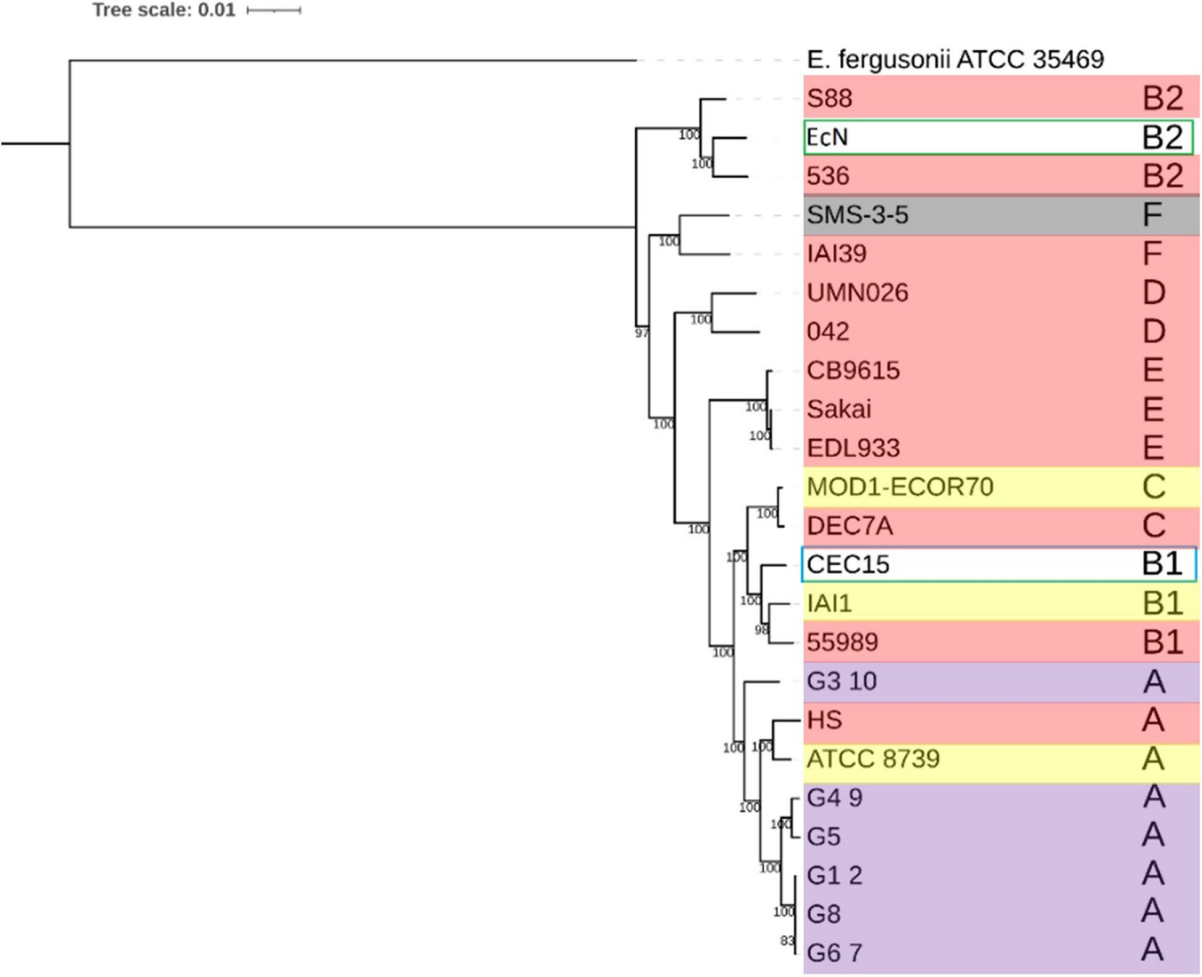
Phylogenomic tree of *Escherichia coli* strains. The phylogenomic analysis was based on 1,000 single-copies genes shared among all the strains. CEC15 and EcN strains are highlighted by a blue and a green box, respectively. Strains highlighted in red are pathogenic strains, while highlights in yellow and gray indicates commensal and environmental strains, respectively. The strains in purple are from the commercial probiotic Synbioflor2®

A phylogenomic tree was constructed with the two studied strains and representative *E. coli* isolates of phylogroups A, B1, B2, C, D, E, including strains from the commercial probiotic product Symbioflor2^®^, and using 1,000 single-copies genes common to all strains (Fig. 1). The CEC15 and EcN strains were scattered throughout the phylogenetic tree. EcN clustered with *E. coli* S88 and 536, two virulent strains belonging to the B2 phylogroup, while CEC15 was found closely related to the strains IAI1 and 55989, a commensal and a pathogenic enteroaggregative strain, respectively, which belong to the *E. coli* phylogroup B1. This analysis showed the high heterogeneity among *E. coli* strains with phylogroups composed of pathogens, commensal, and probiotics. Moreover, it indicated that an association between phylogroup clusters of *E. coli* strains and probiotic properties could not be found.

### CEC15 genome presented fewer genomic islands and mobile elements than EcN

**Fig. 2:**
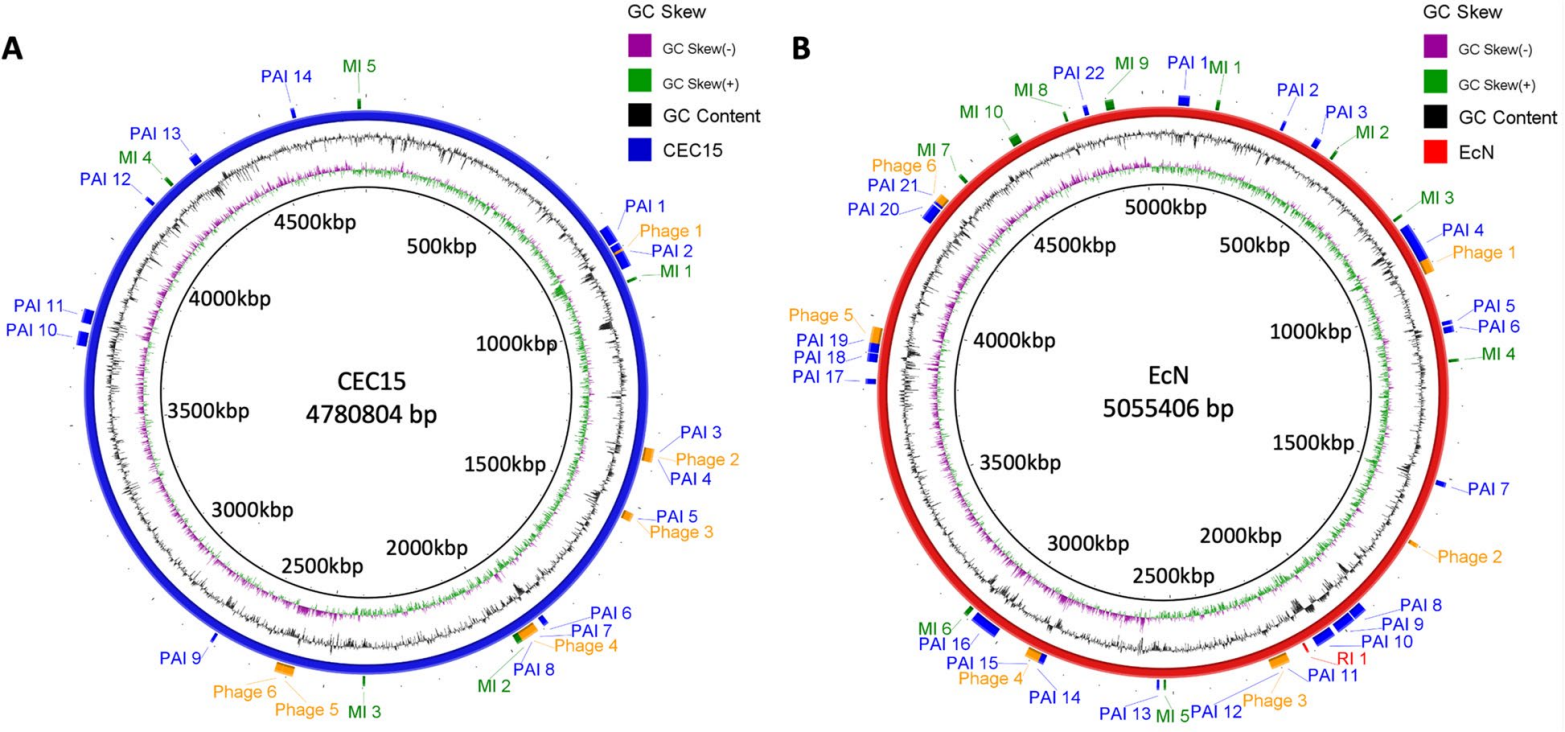
Schematic circular representation of CEC15 (**A**) and EcN (**B**) genomic islands. Pathogenicity Island (PAI), Metabolic Island (MI), Resistance Island (RI), and Prophage regions were found on the genome. Figure generated by BRIG software. Circles, from the inside-out, indicate chromosome size (black circle), the GC skew positive (green) and negative (purple), the GC content (in black indicating higher content outwards and lower content inwards), and the chromosome (blue in figure **A** for CEC15 and red in figure **B** for EcN) with the location of PAI (blue), MI (green), RI (red), and prophage regions (orange)

Prediction analysis revealed the presence of 25 genomic islands (Additional file 1) corresponding to 5 metabolic islands (MI), 14 pathogenicity islands (PAI), and 6 prophage regions (Additional file 2) in the CEC15 genome (Fig. 2A). MI presented lengths ranging from 6 to 18 kb and contained 6 to 23 genes coding for proteins involved notably in the utilization of propanediol, fructose, and mannose (e.g., propanediol utilization (*pdu*) gene cluster, numerous components of PTS sugar transporters). The PAI sizes were larger, with the higher size at 67.8 kb for PAI 2 and the smallest at 7.8 kb for PAI 3. In addition to metabolic functions, PAI2 notably contained genes coding for bacteria competition-related proteins such as colicin immunity domain-containing protein, contact-dependent growth inhibition system immunity protein, and toxin-antitoxin system toxin CbtA family protein. The PAI 1 (48.2 kb) is composed, mainly, of type II secretion system genes, while the PAI 11 (37.1 kb) contains genes from type VI secretion system. The PAI 10 (37.4 kb) contains most genes related to flagella production and assembly, followed by PAI 13 (923.1 kb) that contains genes for fimbriae production. Among the prophage regions found, 3 were predicted to be intact: regions 2 (36 kb), 4 (48.7 kb), and 5 (30.1 kb) that belong to the viral families *Myoviridae*, *Podoviridae*, and *Siphoviridae*, respectively. Note that some PAI and prophage regions overlapped. Prophage region 1 can be found inside PAI 2 while phage region 2 merges with PAI 3 and 4, and phage region 3 merges with PAI 5 almost completely. The large phage region 4 contains the PAI 7 and 8. Those PAI that were found inside prophage regions are mostly composed of transposase genes.

The EcN genome contained more genomic islands than CEC15 with 10 MI, 22 PAI, 1 resistance island (RI), and 6 prophage regions (Fig. 2B) (Additional files 3 and 4). The EcN MI ranged from 6.3 kb to 27 kb and contained mainly genes related to the transport and metabolism of a variety of carbohydrates. The EcN 6.7 kb resistance island is composed of 7 genes, notably one coding for the SMR family multidrug efflux protein EmrE that confers resistance to a wide range of toxic compounds [38]. As for the PAI, besides the large number of islands found, they also have a wide array of sizes ranging from 5.6 kb to 135.6 kb. Many of these PAI contain genes of type II and VI secretion systems, a variety of transposases (IS66, ISl3, ISL100, ISl3, IS21, and IS3), adhesion proteins, iron-binding proteins, and genes encoding proteins associated with antibiotic resistance. An important PAI to be mentioned is the EcN PAI 9 (54.7 kb in size), which contains the biosynthetic gene cluster that produces colibactin, a secondary metabolite that induces DNA double-strand breaks leading to genotoxic effects. None of those genes are found on the CEC15 genome. Of the 6 prophage regions on the EcN genome, 2 were intact (phage region 3 [52.8 kb] and 4 [39.9 kb]), both *Siphoviridae* and these prophage regions merge with genomic islands. The prophage region 3 contains 2 PAI (PAI 11 and PAI 12), while prophage region 4 contains partially the PAI 14 and the whole PAI 15. As for the incomplete prophage regions, prophage region 1 is located completely inside PAI 4, while prophage regions 5 and 6 have some degree of overlapping with PAI 19 and 21, respectively. The prophage region 2 has no overlapping with any PAI. Those PAI contained or overlapping with prophage regions are mainly composed by iron-binding genes, transposases, and metal transport systems.

Analysis of transposable elements, insertion sequences (IS), by the ISSaga tool [39] found 21 complete transposase genes in the CEC15 genome (Additional file 5), from which 9 are present in genomic islands (PAI 2, PAI 7, PAI 11, and PAI 13). EcN has over twice more of transposases genes (48) than CEC15 (Additional file 6), from which 38 were found on PAI (PAI 4, PAI 8, PAI 9, PAI 16, PAI 18, PAI 19, PAI 20, PAI 21, and PAI 22). The CEC15 transposases were characterized in four families (IS3, ISAs1, ISNCY, and ISS66), the IS66 being the most abundant, and, for EcN, into 11 families (IS1, IS110, IS200, IS21, IS3, IS30, IS4, IS630, IS66, ISL3, and ISNCY), IS3 being the most abundant. The majority of IS in the CEC15 genome surrounds sugar metabolism-related genes with 4 IS from the ISS66 family enclosing a phosphotransferase system (PTS) sugar transport cluster, and 2 IS nearby phage regions. The EcN’s IS are, in their majority, surrounding transport-related genes, in addition to four important gene clusters (sialic acid catabolizing gene cluster, flagellar hook-associated protein cluster, salmochelin biosynthesis cluster, and ferric citrate ABC cluster), a few antibiotic resistance genes, and type II and IV toxin/anti-toxin genes. A more detailed superposition of genomic features (PAI, MI, RI, prophages, IS, and antibiotic related genes) of CEC15 and EcN can be found on Additional files 7 and 8, respectively.

### Most antibiotic resistance genes present did not translate to resistance phenotype in CEC15

Forty-five genes coding for proteins potentially related to antibiotic resistance were found in the CEC15 genome by aligning against the Comprehensive Antibiotic Resistance Database (CARD) [40] (Additional file 9). These genes are classified into three resistance mechanisms: antibiotic efflux (*n* = 37), antibiotic target alteration/protection (*n* = 5), and antibiotic inactivation (*n* = 3). The antibiotic classes comprised by these genes are mostly fluoroquinolones, β-lactams, macrolides, glycopeptides, and aminoglycosides. The EcN genome, similarly, presented 44 genes potentially related to antimicrobial resistance (Additional file 10), most of them coding for antibiotic efflux mechanisms (*n* = 38). Four EcN genes were related to antibiotic target alteration/protection, and 2 for antibiotic inactivation. These genes promote resistance to different classes of antibiotics, including aminoglycosides, β-lactams, tetracyclines, fluoroquinolones, macrolides, and glycopeptides.

The distance between antibiotic resistance-related genes and IS is important to evaluate the possibility of genetic transfer to other strains. CEC15 has 12 genes that are < 30 kb distance from an IS gene (*mdtM*, *pmrF*, *evgS*, *evgA*, *emrK*, *emrY*, *eptA*, *mdtE*, *ugd*, *mdtF*, *gadW*, and *gadX*), while EcN has only 6 within the same criteria (*mdtM*, *bacA*, *pmrF*, *ugd*, *cpxA*, and *tolC*) (Additional file 9 and 10, respectively), which are related to resistance to fluoroquinolones, tetracycline, and polymyxin in CEC15 and fluoroquinolones, lincosamides, bacitracin, polymyxin, aminoglycosides, penam, and tetracycline in EcN.

Both strains were submitted to antibiotic susceptibility testing using the disc-diffusion method with antibiotics from nine different classes (Table 1). CEC15 and EcN strains showed susceptibility to most antibiotics but were resistant to erythromycin. The strain EcN showed additional resistance to kanamycin, according to Clinical and Laboratory Standards Institute (CLSI) standards, and to gentamicin, tobramycin, and fosfomycin, according to European Committee on Antimicrobial Susceptibility Testing (EUCAST) standards. Both strains showed intermediate resistance to streptomycin, ampicillin, and ciprofloxacin. Note that the beta-lactamase coding gene *ampC* was found on both strains.

**Table 1:** The antibiotic sensibility of *E. coli* strains (disc-diffusion method)

Antibiotic class	Antibiotic (CODE/µg)	CEC15			EcN		
		halo (mm)	CLSI result	EUCAST result	halo (mm)	CLSI result	EUCAST result
Penicillin	Ampicillin (AMP/10)	15	I	S	16	I	S
	Oxacillin (OXA/5)	0	R	R	0	R	R
Quinolones	Ciprofloxacin (CIP/5)	25	I	S	24	I	ATU
	Chloramphenicol (CHL/30)	25	S	S	26	S	S
	Norfloxacin (NXN/10)	25	S	S	24	S	S
	Nalidixic acid (NAL/30)	19	S	n.d.	20	S	n.d.
Macrolides	Erythromycin (ERY/15)	13	R	n.d.	10	R	n.d.
Aminoglycosides	Gentamicin (GMI/15)	20	S	S	15	S	R
	Kanamycin (KMN/30)	18	S	n.d.	13	R	n.d.
	Streptomycin (SMN/10)	12	I	n.d.	12	I	n.d.
	Tobramycin (TMN/10)	19	S	S	14	I	R
Tetracyclines	Tetracycline (TET/30)	19	S	S	20	S	S
Lincosamides	Lincomycin (LCN/15)	0	R	R	0	R	R
	Clindamycin (CMN/2)	0	R	R	0	R	R
Phosphonic antibiotics	Fosfomycin (FSF/50)	30	S	S	23	S	R

*S* susceptible, *R* resistant, *I* intermediate, *ATU* area of technical uncertainty, *n.d* not described

Despite the high number of genes related to resistance to fluoroquinolones and tetracycline (19 and 11 genes, respectively in both strains), CEC15 and EcN showed sensitivity to all antibiotics tested from these classes.

### CEC15 and EcN did not present hemolytic activity

**Fig. 3:**
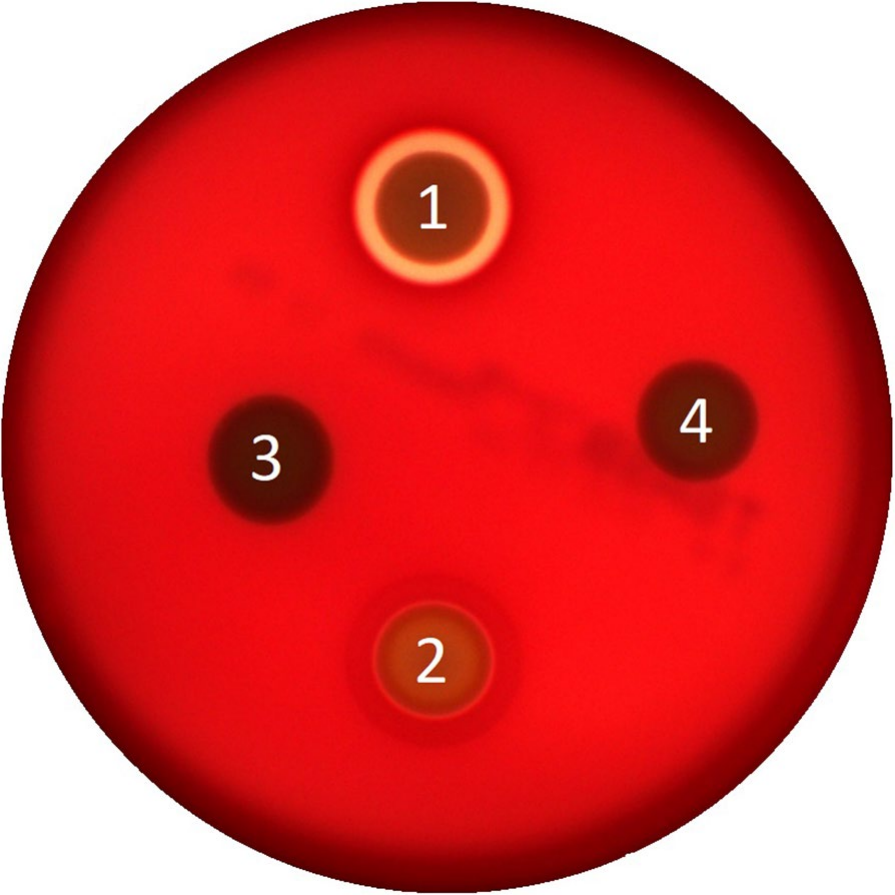
Hemolytic activity assay of *E. coli* strains. Strains *Staphylococcus aureus* IT2 (1), *S. aureus* Bk (2), CEC15 (3), and EcN (4) were spotted on sheep-blood agar and incubated overnight, the presence of a halo was observed for the two control strains (1 and 2) but not for the tested strains in this study (3 and 4)

Four hemolysis related genes were found in both CEC15 and EcN genome: genes coding for a ShlB/FhaC/HecB family hemolysin secretion/activation protein, a hemolysin III family protein, a hemolysin family protein, and hemolysin HlyE. Moreover, EcN presented the hemolysin expression modulator *hha* gene. The ShlB/FhaC/HecB family hemolysin secretion/activation protein-encoding gene is found inside PAI for both strains (PAI 2 and 18 for CEC15 and EcN, respectively), and *hha* gene is found in PAI 4 for EcN, remaining genes are found elsewhere in the chromosome. The hemolytic activity of strains CEC15 and EcN was therefore evaluated on sheep-blood agar, with the two *S. aureus* strains Bk and IT2 as a control for α- and β-hemolysis, respectively. Complete hemolysis was observed for strain IT2 (Fig. 3, spot 1) with a yellow halo corresponding to a β-hemolytic activity, whereas strain Bk only resulted in partial degradation of erythrocytes leading to a greenish halo, which is characteristic of α-hemolytic activity (Fig. 3, spot 2). No halo was observed for strains CEC15 and EcN showing their inability to degrade erythrocytes (Fig. 3, spots 3 and 4 respectively).

### Metabolic profile of CEC15 revealed exclusive pathways for GABA and sugar production, amino acid metabolism, and xenobiotics degradation

The different number of MI between the two strains prompted us to examine their metabolic abilities. As expected, these strains share most metabolic pathways (KEGG [Kyoto Encyclopedia of Genes and Genomes] modules) identified by the BlastKOALA [41] analysis. The strains share, in total, 100 complete metabolic modules, while 5 modules, found exclusively in the CEC15 genome, are involved in polyamine biosynthesis (synthesis of gamma-aminobutyric acid, GABA, from putrescine), aromatic amino acid metabolism (homoprotocatechuate degradation), polyketide sugar unit biosynthesis (dTDP-L-rhamnose biosynthesis), and two aromatics (xenobiotics) degradation modules (phenylacetate degradation and trans-cinnamate degradation). No exclusive modules were found on EcN (Additional file 11).

Both strains have the machinery necessary to produce 6 from the 8 essential amino acids (lysine, threonine, isoleucine, methionine, phenylalanine, and tryptophan) and other 7 non-essential amino acids (arginine, cysteine, histidine, proline, serine, tyrosine, and glutamate), and cofactors and vitamins, especially from the B group (pantothenate, biotin, pyridoxal-p, and riboflavin). The predicted gene repertoires of complete pathways for sugar utilization in the CEC15 and EcN genomes allow the metabolism of galactose, fructose, xylulose, ribulose, ribose, erythrose, lactose, ascorbate, glycogen, and starch as primary carbon source. Another gene class with an important role on the carbohydrate metabolism is the group of PTS sugar transport systems, which are present in large amount in both genomes (59 and 64 genes for CEC15 and EcN, respectively), allowing the entry of sugars into the cell to be metabolized. The genome of both strains also comprises genes involved in two terpenoids biosynthesis, C5 isoprenoid and C10-C20 isoprenoid (Additional file 11).

### CEC15 demonstrated high fitness on simulated human digestion

Twenty-five genes related to acid tolerance were found, 23 shared among both strains and 2 exclusives of CEC15 (peroxide/acid resistance protein YodD and YceO family protein) (Table 2). The highly associated acid resistance genes from the glutamate decarboxylase family (GAD family) [42] and the acid stress response sigma factor RpoS [43] were found in the genome of both strains, which could indicate a high survival rate for both in the gastric environment.

**Table 2:** Acid-resistance proteins found on the genome of CEC15 and EcN

**Locus tag**		**Gene**	**Product**
CEC15_000207	EcN_000211	*gadA*	glutamate decarboxylase
CEC15_000208	EcN_000212	*gadX*	acid resistance transcriptional activator GadX
CEC15_000213	EcN_000217	*gadE*	acid resistance transcriptional activator GadE
CEC15_000215	EcN_000219	*hdeA*	acid-activated periplasmic chaperone HdeA
CEC15_000216	EcN_000220	*hdeB*	acid-activated periplasmic chaperone HdeB
CEC15_000474	EcN_000513	*yhcN*	peroxide/acid stress response protein YhcN
CEC15_001489	EcN_001445	*oxc*	oxalyl-CoA decarboxylase
CEC15_001491	EcN_001447	*yfdE*	CoA:oxalate CoA-transferase
CEC15_001977		*yodD*	peroxide/acid resistance protein YodD
CEC15_002333	EcN_002372	*asr*	acid resistance repetitive basic protein Asr
CEC15_002338	EcN_002377	*clcB*	voltage-gated ClC-type chloride channel ClcB
CEC15_002404	EcN_002437	*ydeO*	acid stress response transcriptional regulator YdeO
CEC15_002411	EcN_002444	*gadB*	glutamate decarboxylase
CEC15_002412	EcN_002445	*gadC*	acid resistance gamma-aminobutyrate antiporter GadC
CEC15_002520	EcN_002529	*ldhA*	D-lactate dehydrogenase
CEC15_002734	EcN_002690	*ychM*	C4-dicarboxylic acid transporter DauA
CEC15_002784	EcN_002738	*ariR*	biofilm/acid-resistance regulator AriR
CEC15_002800	EcN_002809	*phoQ*	two-component system sensor histidine kinase PhoQ
CEC15_002870		*yceO*	YceO family protein
CEC15_002912	EcN_003035	*ymdF*	general stress protein
CEC15_003598	EcN_003686	*yagU*	YagU family protein
CEC15_003764	EcN_003863	*clcA*	H(+)/Cl(-) exchange transporter ClcA
CEC15_004198	EcN_004368	*adiC*	arginine/agmatine antiporter
CEC15_004546	EcN_004770	*ilvD*	dihydroxy-acid dehydratase
CEC15_001098	EcN_001127	*rpoS*	RNA polymerase sigma factor RpoS

**Fig. 4:**
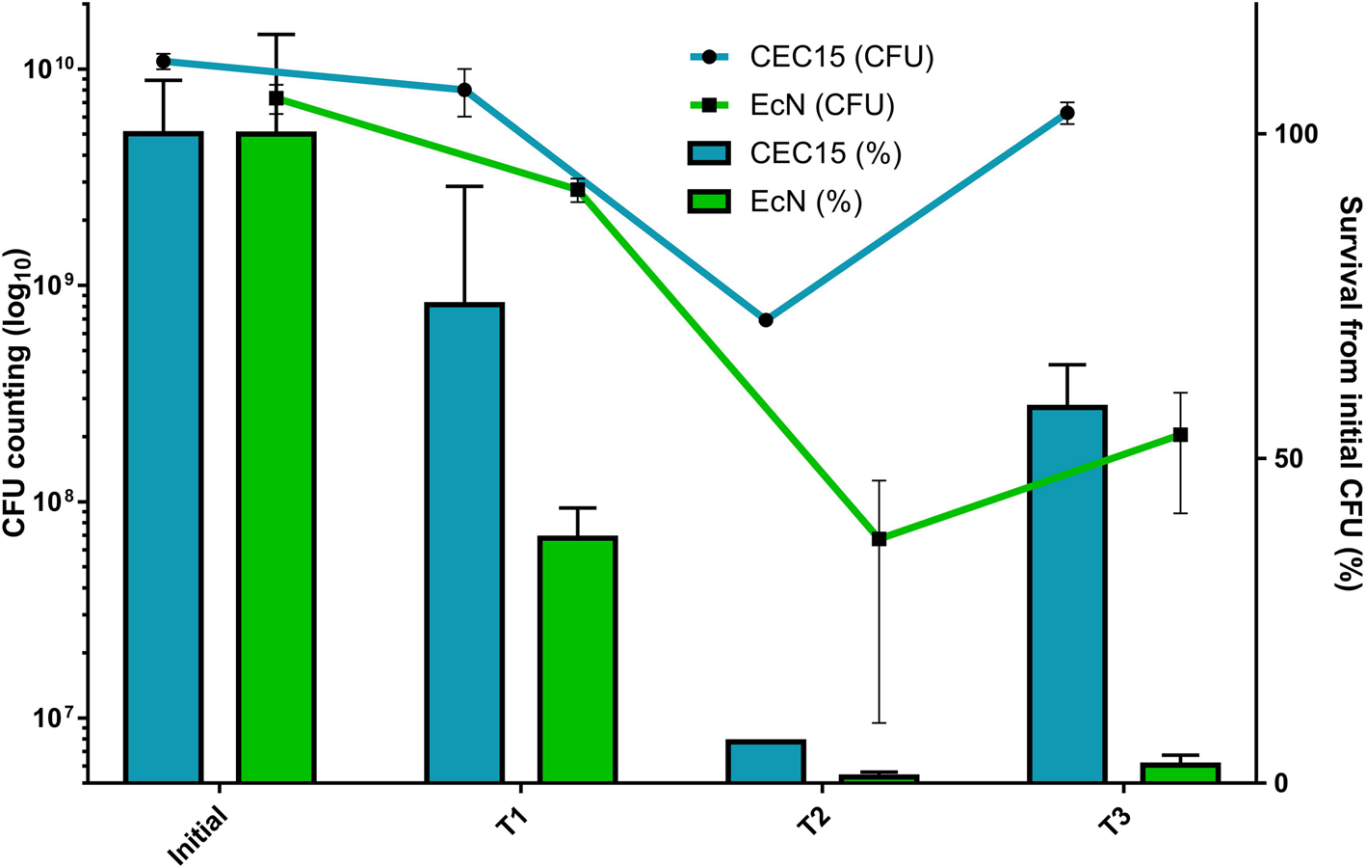
Bacterial survival in the simulated human digestive tract. Both strains were submitted to an artificial digestion process and, at each step, aliquots were collected to estimate the quantity of viable bacteria. CFU counting were made before the experiment begins (Initial), at the start of gastric phase (pH adjusted to 3 - T1), at the end of gastric phase and beginning of intestinal phase (120 min in pH 3 - T2), and at the end of intestinal phase (pH restored to 7 and 120 min incubation - T3). The lines represent the CFU count in each step of the digestion processes while the bars represent the viability in percentage relative to the initial CFU. Data are expressed as mean and standard deviation of three independent experiments

To evaluate this hypothesis, the viability of the two strains was assessed in gastrointestinal conditions using a simulated human digestion protocol. Both strains underwent a considerable loss of viability, just after the pH was adjusted to 3, with a survival rate of 73.7% (± 0.08, *p*=0.016) for CEC15 and 37.71% (± 0.15%, *p*<0.0001) for EcN (Fig. 4, T1). After 120 min of incubation at pH 3 and in the presence of pepsin, simulating the gastric environment, 6.3% (± 0.001%, *p*<0.0001) of the initial concentration of CEC15 were still viable, against 0.91% (± 0.01%, *p*<0.0001) for EcN (Fig. 4, T2). After changing to the intestinal environment (pH 7, pancreatin, and bile salts) and incubating for another 120 min, the CEC15 strain presented a considerable recovery of colony forming units (CFU), restoring its viability to 57.85% (± 0.07%, *p*=0.0004) of the initial concentration, while EcN CFU was maintained at 2.77% (± 0.02%, *p*<0.0001) (Fig. 4, T3), which represents no significative difference with the previous phase (EcN T2 vs T3, *p*=0.9939) (Fig. 4). These results indicate that the CEC15 strain is likely more fit to survive the stress promoted by the gastrointestinal tract environment, being more able to thrive in those conditions than the EcN strain.

### High adhesion rate of CEC15 can be associated to the presence of fimbriae and pili

According to the SPAAN software [44], 84 genes of CEC15 and 89 of EcN, six from each are duplicated genes (Additional file 12) were predicted with a high probability profile (score> 0.8) to code for adhesins. A total of 33 genes were found exclusively on CEC15 genome, against 32 on EcN. From these exclusive gene products, CEC15 presents 13 fimbriae proteins, 2 flagella proteins, 9 transport proteins, and 4 phage related proteins. EcN, on the other hand, possess 7 fimbriae proteins, 5 transport proteins, and 3 phage related proteins. Among the predicted CEC15 adhesin genes, 29 are related to fimbriae/pili proteins (34%), 18 to porins/transporters (21%), and 8 to flagella proteins (10%). A similar categorical distribution of adhesins was observed for EcN: 30% of fimbriae/pili (*n* = 27), 13% of porins/transporters (*n* = 15), and 7% of flagella (*n* = 6). The 5 highest-scored genes on CEC15 are related to contact-dependent inhibition toxin CdiA, type 1 fimbria D-mannose specific adhesin FimH, lateral flagellin LafA, exopolysaccharide production protein YjbE, and type 1 fimbrial major subunit FimA, while for EcN we found contact-dependent inhibition effector tRNA nuclease, type 1 fimbria D-mannose specific adhesin FimH, phase-variable autotransporter adhesin UpaE, DUF823 domain-containing adhesin, and F1C fimbria minor subunit FocG.

**Fig. 5:**
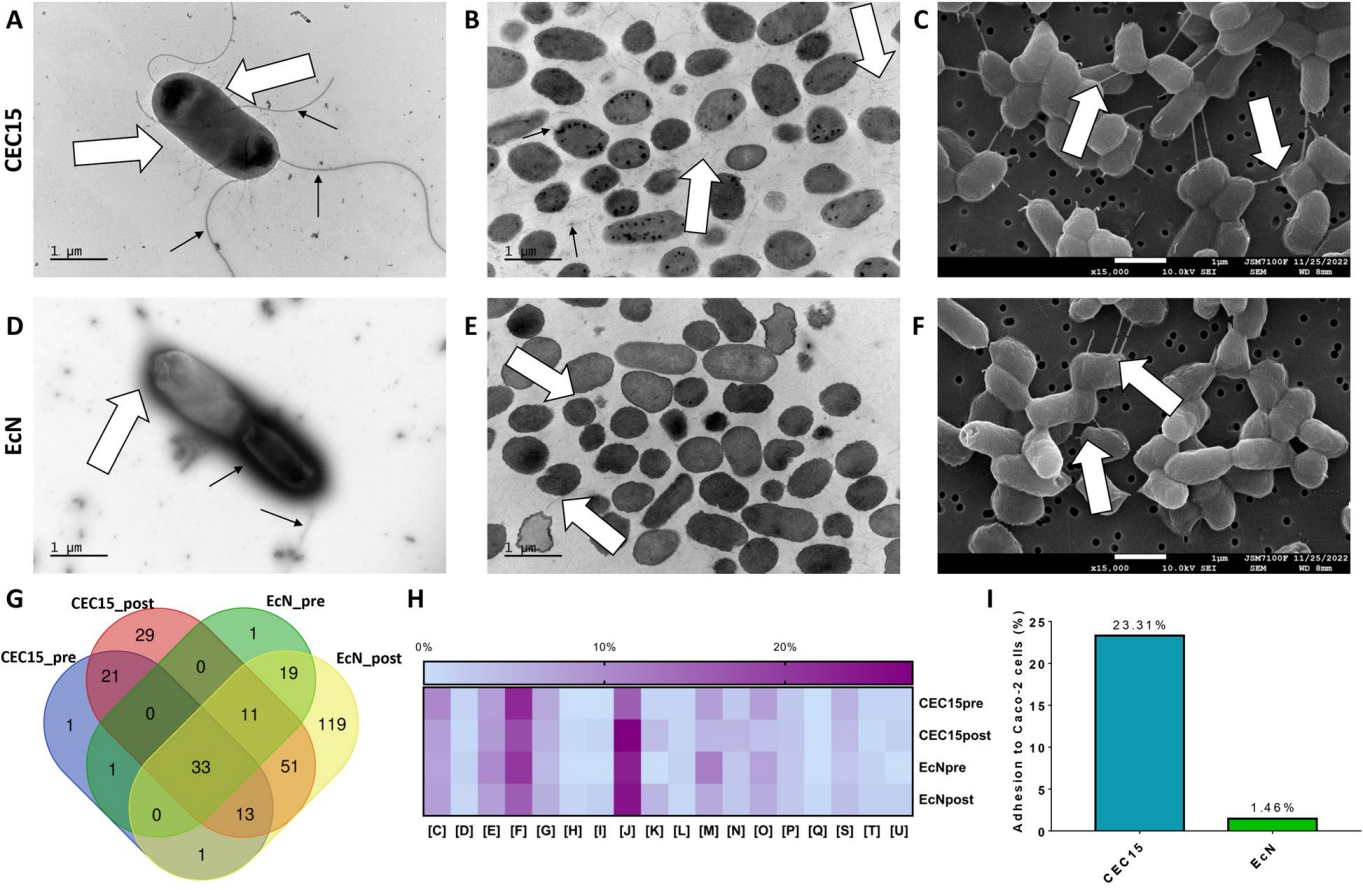
Adhesive profile of CEC15 and EcN strains. The presence of fimbriae/pili and flagella in CEC15 and EcN strains could be confirmed by Transmission electron microscopy of fresh (**A** and **D**) and fixated (**B** and **E**), and by scanning electron microscopy (SEM) of fixated samples (**C** and **F**) of CEC15 and EcN, respectively. A significant quantity of proteins were found on sheared samples of both strains, about half of them being shared between strains (**G**). The heatmap (**H**) present the percentage of COG-classified proteins presented in each condition, according to the code: [C] Energy production and conversion; [D] Cell cycle control, cell division, chromosome partitioning; [E] Amino Acid metabolism and transport; [F] Nucleotide metabolism and transport; [G] Carbohydrate metabolism and transport; [H] Coenzyme metabolism; [I] Lipid metabolism; [J] Translation; [K] Transcription; [L] Replication and repair; [M] Cell wall/membrane/envelop biogenesis; [N] Cell motility; [O] Post-translational modification, protein turnover, chaperone functions; [P] Inorganic ion transport and metabolism; [Q] Secondary metabolites biosynthesis, transport and catabolism; [S] Function Unknown; [T] Signal Transduction; [U] Intracellular trafficking and secretion. The effectiveness of these adhesins were tested by adhesion assay on Caco-2 cells (**I**) were CEC15 presented a better adhesive profile (23.31%) than EcN (1.46%). White arrows indicate the presence of fimbriae/pili. Black arrows indicate flagella. Scale in all pictures is equivalent to 1µm and the pictures were taken on amplification of 30,000 for TEM and 15,000 for SEM

The presence of surface appendages like fimbriae/pili and flagella was confirmed by electron microscopy in both strains. The scanning electron microscopy (SEM) and transmission electron microscopy (TEM) images (Fig. 5A-F) suggest that the CEC15 strain expresses more fimbriae/pili (white arrows) on its surface than the EcN strain. To study the expression of these proteins on the surface of both strains, a mechanical shearing of overnight still culture was performed. The extracted proteins (Additional file 13) were digested by in-gel trypsinolysis and identified through MS/MS mass spectrometry.

A variety of proteins were found following shearing of the bacteria. The identification of proteins proceeded with samples pre-shearing (only resuspended in phosphate-buffered saline [PBS] buffer) and post-shearing (resuspended in PBS and then blended, 5 min, max speed). Regarding pre-shearing samples, a total of 70 and 65 proteins were detected for CEC15 and EcN, respectively, 34 of those shared among both strains, while after shearing, the quantity of proteins detected increased to 158 on CEC15 and to 247 for EcN, with 108 being shared (Fig. 5G) (Additional file 14). A total of 50 proteins were exclusive to CEC15, among those, 1 (autotransporter outer membrane beta-barrel domain-containing protein) were exclusive to pre-shearing CEC15, 29 on post-shearing CEC15 (notably flagellar hook protein FlgE, flagellar filament capping protein FliD, flagellar hook-associated protein FlgL, type 1 fimbria chaperone FimC, type 1 fimbria D-mannose specific adhesin FimH, and type 1 fimbria minor subunit FimG), and 20 shared on pre- and post-shearing (notably flagellin FliC). EcN, on the other hand, presented 139 exclusive proteins, 1 (peptidoglycan-associated lipoprotein Pal) on pre-shearing sample, 119 on post-shearing samples (notably flagellar hook protein FlgE, autotransporter adhesin Ag43, Ag43/Cah family autotransporter adhesin, flagellar hook-associated protein FlgK, F1C fimbrial major subunit FocA, F1C fimbrial protein subunit FocH), and 19 shared on pre- and post-shearing (notably FliC/FljB family flagellin). Based on the emPAI, we can infer that flagellin FliC is the main protein on CEC15 samples, FliC/FljB family flagellin on EcN pre-shearing, and F1C fimbrial major subunit FocA on EcN post-shearing (Supplementary Table S12).

The identified proteins were categorized according to their COG (Clusters of Orthologous Genes) classes. All samples had a high prevalence of translation proteins [J] (15.58% and 27.05% for CEC15 and 23.18% and 24.62% for EcN, pre- and post-shearing respectively) and Nucleotide metabolism and transport [F] (22.07% and 17.64% for CEC15 and 20.28% and 15.53% for EcN, pre- and post-shearing respectively), what could indicate cell lysis, as they are represented mainly by ribosomal proteins and enzymes. An important COG class for adhesion proteins is the Cell wall/membrane/envelop biogenesis [M] category, and it represented 7.79% and 4.70% for CEC15, and 11.59% and 6.43% for EcN (pre- and post-shearing, respectively) (Fig. 5H).

Based on the previous results, the adhesion of strains CEC15 and EcN to the human Caco-2 intestinal epithelial cell line was investigated. CEC15 exhibited the highest adhesion ability (~23%) on Caco-2 cells when compared to EcN strain (~1.5%). (Fig. 5I).

### More bacteriocins clusters were detected on EcN than CEC15

Three gene clusters related to the synthesis of bacteriocins were found in EcN genome: a cluster coding for genes involved in the synthesis of bottromycin, an inhibitor of protein synthesis that blocks aminoacyl-tRNA binding; a cluster coding for microcin production and transport, a channel-forming bacteriocin active against enterobacteria; and a cluster coding for colicin-E9 production and transport, a polypeptide toxin with endonuclease activity against *E. coli* strains and closed-related bacteria. The EcN genome presented all three gene clusters, while only the bottromycin-encoding cluster was found in the CEC15 genome (Additional file 15).

### CEC15 promoted modulation of immune- and barrier-related gene expression in Caco-2 cells

**Fig. 6:**
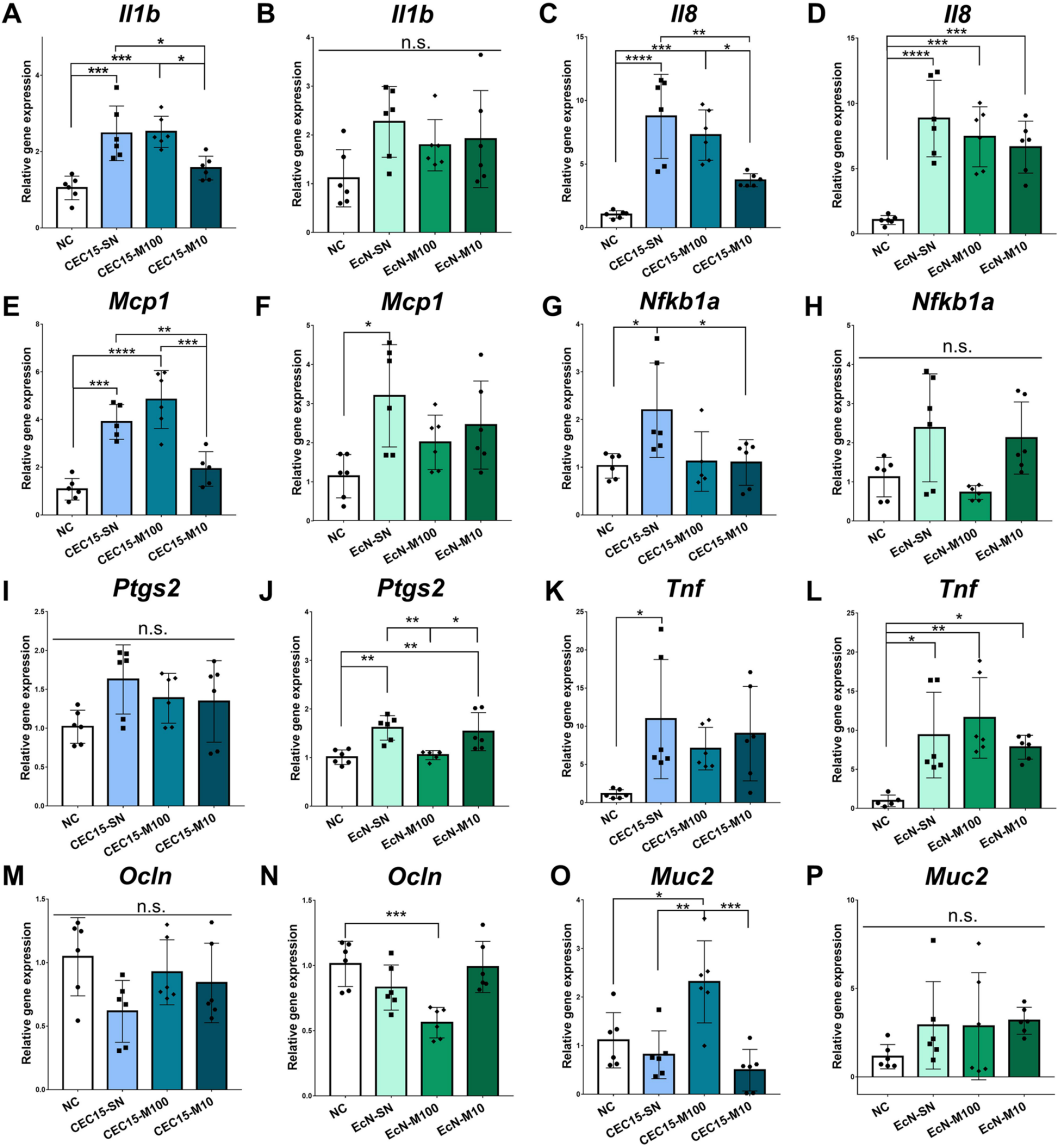
Modulation of immunoregulatory and barrier-related genes expression in Caco-2 cells. The relative gene expression of genes related to immunomodulation and intestinal barrier (*Il1b* (**A** and **B**), *Il8* (**C** and **D**), *Mcp1* (**E** and **F**), *Nfkb1a* (**G** and **H**), *Ptgs2* (**I** and **J**), *Tnf* (**K** and **L**), *Ocln* (**M** and **N**), and *Muc2* (**O** and **P**)) on CEC15- and EcN-treated cells, respectively, were evaluated with the *Gapdh*, *B2m*, and *Hprt1* genes as reference (2^- ΔΔct^). Statistical analyses were performed by One-way ANOVA with Tukey’s post-test on GraphPad Prism 7.0. * *p*<0.05; ** *p*<0.01; *** *p*<0.001; **** *p*<0.0001. NC: negative control; CEC15-SN: CEC15 supernatant; CEC15-M100: CEC15 treatment at MOI 100; CEC15-M10: CEC15 treatment at MOI 10; EcN-SN: EcN supernatant; EcN-M100: EcN treatment at MOI 100; EcN-M10: EcN15 treatment at MOI 10

The ability of both strains to modulate the expression of intestinal epithelial cell genes coding for key factors of immunoregulation and epithelial integrity was evaluated. For this purpose, Caco-2 cell monolayers were incubated with the bacterial supernatants or with heat inactivated bacterial cells of both strains and the expression of Caco-2 genes was evaluated after 24 h of treatment. CEC15 strain appeared to be more immunomodulatory than EcN. Indeed, CEC15 supernatant and/or inactivated CEC15 cells increased the expression of 6 genes, (*Il1b, Il8, Mcp1, Nfkb1a, Tnf*, and *Muc2*), while EcN only modulated 5 genes, increasing the expression of *Il8, Mcp1, Tnf,* and *Ptgs2*, and reducing the expression of *Ocln* among those tested. The remaining genes were not altered by any of the treatments (Additional file 16). Indeed, among the 6 barrier-related genes tested, only heat treated CEC15 at multiplicity of infection (MOI) of 100:1 induced the expression of *Muc2*, while only heat treated EcN at MOI of 100:1 lowered the expression of *Ocln*. In addition, *Ptsg2* expression, which in the colonic environment is highly associated with the promotion of colorectal carcinoma, was only induced by EcN (Fig. 6).

### CEC15 showed better intestinal protection against 5-FU-induced mucositis than EcN

We evaluated the impact of a high-dosage daily administration of strains CEC15 and EcN, and of their anti-inflammatory and protective effects, in the context of 5-FU-induced intestinal mucositis in a BALB/c mice model.

**Fig. 7:**
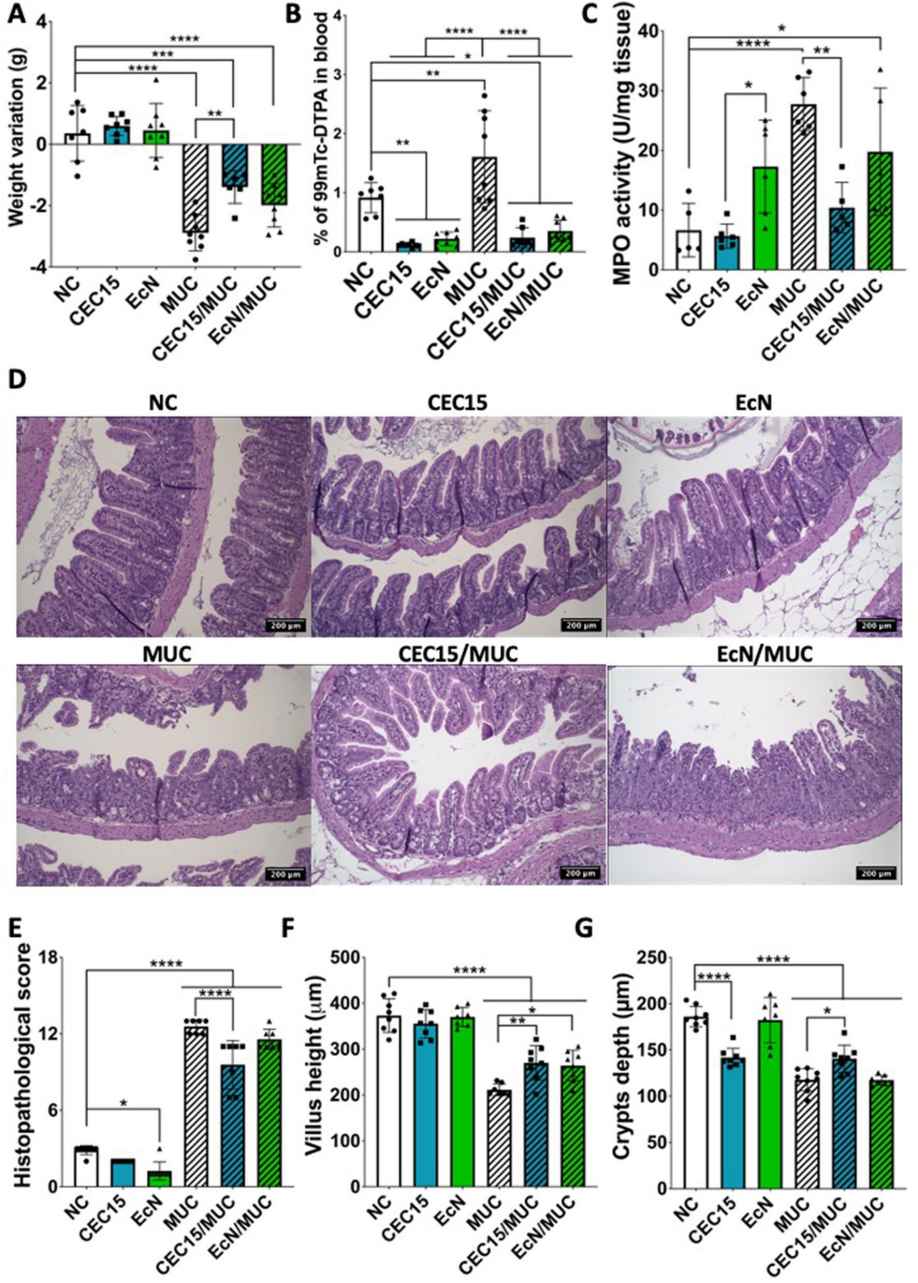
Clinical and histopathological aspects of *E. coli* strains’ administration. Mice (*n* = 8) were administered either sterile PBS, CEC15 (10^10^ CFU/mice/day) or EcN (10^10^ CFU/mice/day) for 12 days with administration of 5-FU (300 mg/kg) or PBS on the day 10. The results above show the weight variation (day 10-13) (**A**) and the morphological characteristics, such as intestinal permeability (**B**) and tissue neutrophilic infiltration (**C**). The structural damage caused by the 5-FU administration and the partial protection promoted by CEC15, as well as the unmodified morphology on the control groups and of the EcN treatment after mucositis induction can be observed on the slides (**D**), dyed with hematoxylin and eosin (Magnification of 20X). The histopathologic inflammatory scoring based on villous atrophy, rupture of the surface enterocyte borders, depletion of calyceal cells, loss of crypt architecture, destruction of crypt cells, abscess formation in the crypts, infiltration of lymphocytes and polymorphonuclear cells, dilation of capillaries and lymphatic vessels, and thickening with edema formation in the submucosa and external muscle layers. Histological features were scored on a scale of 0 (average) to 3 (max damage), and points were summed for each animal accordingly (**E**), villus height (**F**), and the depth of the crypts (**G**) were measured from these slides. Statistical analyses were performed by One-way ANOVA with Tukey’s post-test on GraphPad Prism 7.0. * *p*<0.05; ** *p*<0.01; *** *p*<0.001; **** *p*<0.0001. NC: negative control; CEC15: healthy CEC15-treated; EcN: healthy EcN-treated; MUC: mucositis control; CEC15/MUC: mucositis CEC15-treated; EcN/MUC: mucositis EcN-treated

Both strains were administered, as a daily dose of 10^10^ CFU, via gavage, for 12 consecutive days, to healthy animals and to animals with 5-fluorouracil (5-FU)-induced mucositis. During the experimental period, no significant difference in body weight and in food and water intake was found between groups of healthy animals that received either PBS (control group; NC), CEC15 or EcN. The induction of mucositis led to a weight loss of about 3.5 g per animal. Consumption of CEC15 or of EcN did not totally overcome mucositis-related weight loss in animals. However, consumption of the CEC15 strain (CEC15-MUC) was able to partially prevent this weight loss, when compared to the MUC group (Fig. 7A). 5-FU-induced mucositis drastically increased intestinal permeability, as indicated by the increased blood counts of DTPA-^99^Tc by almost 2-fold, in comparison to the NC group. However, both strains prevented this increase in permeability. Moreover, in the absence of 5-FU, they reduced permeability to levels below that of the NC group (Fig. 7B). The neutrophilic infiltration, as indicated by the intestinal myeloperoxidase (MPO) activity, was increased in mucositis animals. Among the tested strains, only CEC15 reduce MPO activity down to a level close to that of healthy animals (Fig. 7C).

The structure of the ileal epithelium can be observed on the HE-stained tissue sections in Fig. 7D. The structural damages caused by 5-FU-induced mucositis were evaluated by histopathological scoring of such sections (Fig. 7E). The analysis showed an extensive damage of the ileal epithelial structure caused by the administration of 5-FU. This damage, however, was attenuated by the administration of CEC15 (Fig. 7E). In line with the observed alterations of the ileal mucosa structure, infiltration of inflammatory neutrophils was quantified by monitoring the MPO activity. This infiltration was prevented by consumption of CEC15 (Fig. 7C). 5-FU administration also affected the height of villi and the crypts depth (Fig. 7F-G). The treatments with CEC15 and EcN were able to reduce the damage on the villi height (Fig. 7F), but only the CEC15 treatment was able to prevent reduction of crypts depth (Fig. 7G).

### CEC15 led to increased microbial diversity in healthy animals and reduced dysbiosis in 5-FU-induced mucositis.

**Fig. 8:**
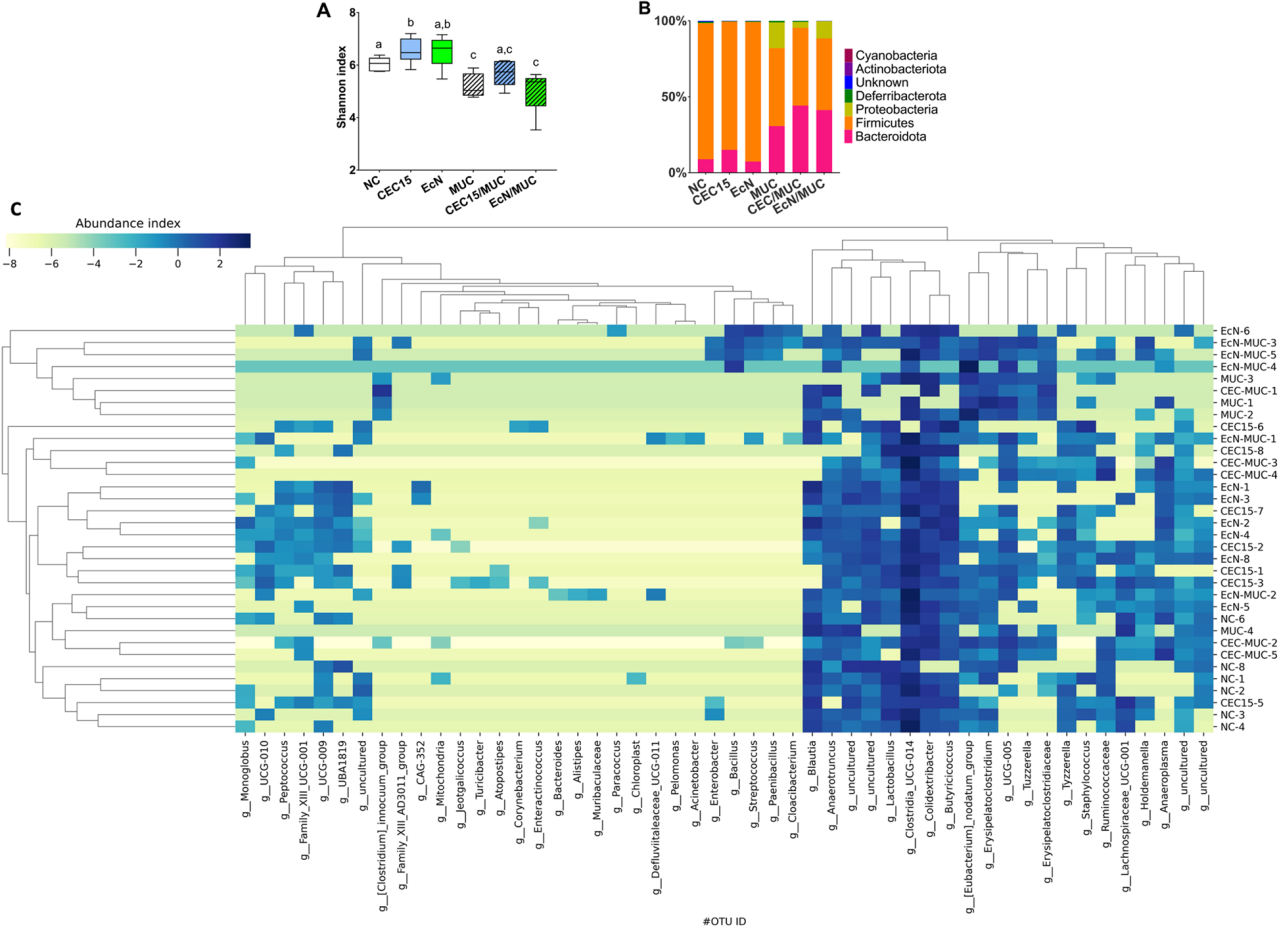
Alpha diversity and abundance of OTU of intestinal bacteria. **A** Alpha diversity shown by Shannon index estimated for each group: NC: negative control (*n* = 6); CEC15: healthy CEC15-treated (*n* = 7); EcN: healthy EcN-treated (*n* = 7); MUC: mucositis control (*n* = 4); CEC/MUC: mucositis CEC15-treated (*n* = 5); EcN/MUC: mucositis EcN-treated (*n* = 5). **B** Relative abundance of intestinal microbiota at the phylum level among the groups. **C** Heatmap analysis of the bacterial genus distribution among the 34 samples based on hierarchical clustering. One-way ANOVA and Bonferroni multiple comparisons test. Different letters indicate significative differences, *p* value < 0.05

We evaluated how the *E. coli* strains modulated the gut microbiota, both in a healthy context, and in the context of 5-FU-induced mucositis. In that aim, feces collected on the last day of the animal experiment above mentioned were analyzed by 16S rRNA amplicon sequencing. The alpha diversity of the groups, represented by the Shannon index, showed statistical difference between the healthy and the mucositis groups (Fig. 8A). CEC15 presented a higher diversity than NC but showed no difference from EcN. 5-FU administration caused a loss of diversity in the intestinal microbiota, demonstrated by the difference between the NC and MUC groups. Interestingly, the treatment with CEC15 (CEC/MUC group) partially protected against loss of diversity. The analysis of the relative abundance in the groups at the phylum level (Fig. 8B) showed no significative difference on abundance in the healthy groups and an imbalance of the microbiota with increasing in abundance of *Bacteroidota* and *Proteobacteria* member in the MUC group samples compared to the NC. Although both CEC15 and EcN treatments reduced the abundance of *Proteobacteria* in the 5-FU groups, the impact of CEC15 was more prominent. The heatmap (Fig. 8C) shows the diversity on the microbiota in the genus level among samples and treatment groups, the genera with high abundance are *Parabacteroides*, *Clostridia*, *Lachnospiraceae*, and *Eubacterium*.

**Fig. 9:**
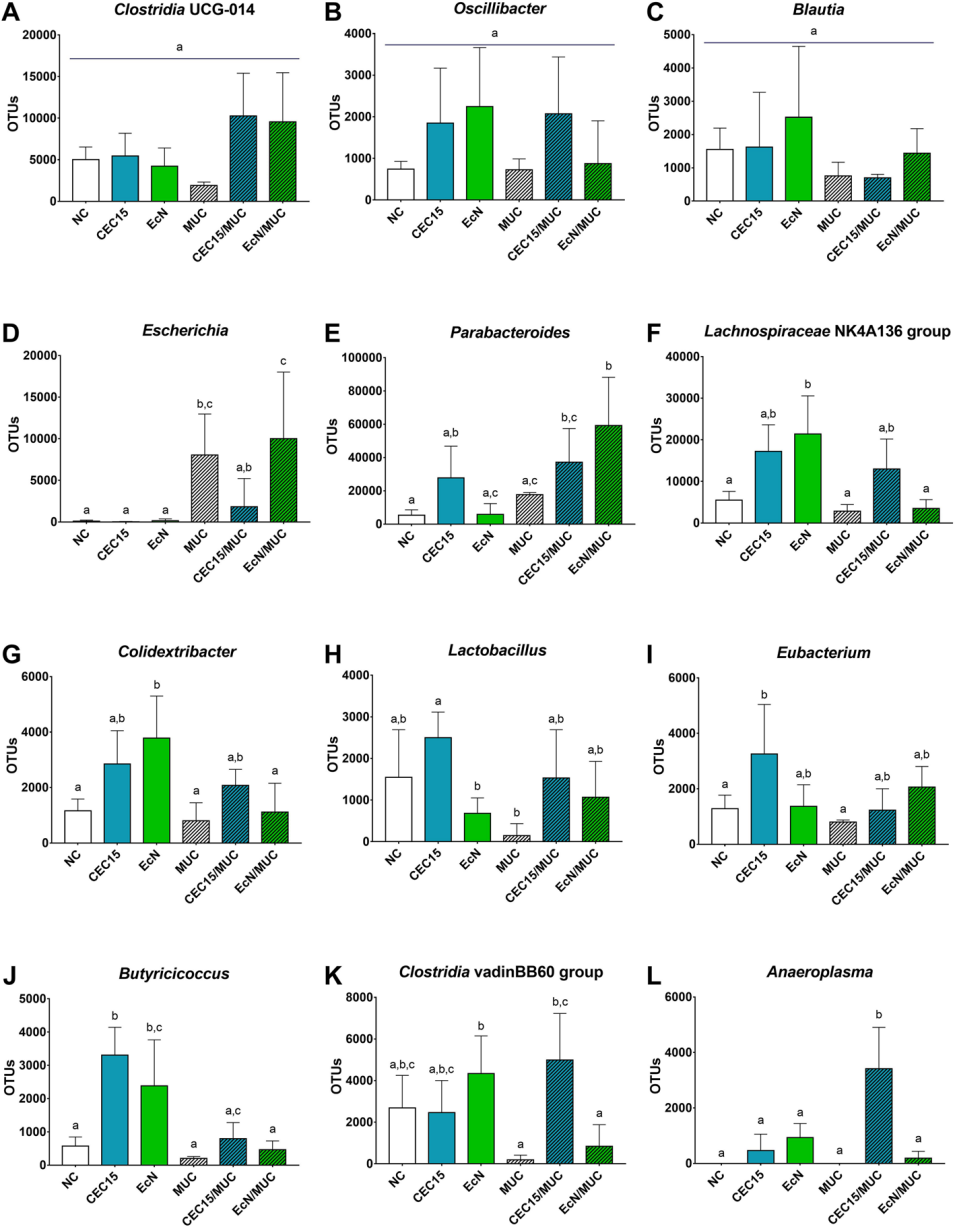
Relative abundance of the main genus of fecal bacteria. **A**–**L** abundances of genus in the feces of mice in different groups. Data are expressed in absolute OTU reads (± S.E.M). Different letters indicate statistical significance (*P* < 0.05; one-way ANOVA and Bonferroni multiple comparisons test). NC: negative control (*n* = 6); CEC15: healthy CEC15-treated (*n* = 7); EcN: healthy EcN-treated (*n* = 7); MUC: mucositis control (*n* = 4); CEC/MUC: mucositis CEC15-treated (*n* = 5); EcN/MUC: mucositis EcN-treated (*n* = 5)

The twelve genera with the highest abundance were compared individually between groups (Fig. 9). Three genera belonging to the *Firmicutes* phylum did not present any significative difference among groups (*Clostridia* UCG-014, panel A; *Oscillibacter*, panel B; *Blautia*, panel C). *Escherichia* (panel D) is the only genus that present significant difference between NC and MUC groups (*p*=0.0356), no difference was see among the healthy groups. *Escherichia* abundance was the same between NC and the CEC/MUC and EcN/MUC groups however, these two groups presented different abundance among themselves (CEC/MUC vs EcN/MUC, *p*=0.0388). Regarding *Parabacteroides* (panel E), CEC15/MUC and EcN/MUC showed a higher abundance in relation to NC (*p*=0.0006), while only EcN/MUC presented a higher abundance than EcN (*p*=0.0007), and MUC (*p*=0.0373). *Lachnospiraceae* (panel F) and *Colidextribacter* (panel G) presented similar pattern, with EcN showing difference from NC (*p*=0.0142; *p*=0.0052), MUC (*p*=0.0079; *p*=0.0025), and EcN/MUC (*p*=0.0113; *p*=0.0044). As for *Lactobacillus* (panel H), CEC15 presented a higher abundance than EcN (*p*=0.0174) and MUC (*p*=0.0025), in the *Eubacterium* genus (panel I) CEC15 presented difference with NC (*p*=0.0408) and MUC (*p*=0.0352). The *Butyricoccus* genus (panel J) was increased in CEC and EcN, presenting difference to NC (*p*=0.0004; *p*=0.0232), MUC (*p*= 0.0006; *p*=0.0190), and the treatments, CEC/MUC (*p*=0.0060 against CEC15), and EcN/MUC (*p*=0.0005; *p*=0.0249). The *Clostridia* vadinBB60 (panel K) group was modulated positively with the treatment with CEC15 on mucositis animals (CEC/MUC vs MUC, *p*=0.0029), which was not seen for the treatment with EcN. Finally, the *Anaeroplasma* genus (panel L) presented no difference among the healthy groups and the MUC group, in the CEC/MUC group, however, the number of OTUs was highly increase (*p*<0.0001 against all groups), the same was not observed for EcN treatment. These results showed the capability of CEC15 and EcN to modulate the gut microbiota composition, including some important genera in healthy animals and in the context of an inflammatory gastrointestinal disease.

## Discussion

Although there are numerous probiotic strains available in the market, there is still a need for new strains with new or enhanced beneficial properties. It is crucial to note that such properties are specific to each strain, and as such, it is of utmost importance to thoroughly identify and characterize any potential new probiotic strain to determine the most beneficial ones [45]. The commensal *Escherichia coli* strain CEC15 has shown promising protective properties in a chronic colitis mouse model [37]. In the present study, CEC15 was assessed for other properties relevant to a probiotic bacterium, using a probiogenomics approach combined with *in vitro* and *in vivo* analyses. We tested its safety, its antibiotic resistance, the presence of pathogenic characteristics, tolerance towards gastrointestinal conditions, adhesion to intestinal cells, immunomodulatory properties, and protective effects in a 5-FU-induced intestinal mucositis mice model. Among the relevant features we identified for a probiotic, we can mention the absence of hemolytic activity, the presence of genes associated with antioxidant properties (e.g., biosynthesis of terpenoids) and ability to modulate the inflammatory process. While some beneficial properties are shared by both strains, others, which are of great value for a probiotic, are specific to the CEC15 strain. In this study, we conducted a comprehensive comparison of CEC15, focusing on its probiotic attributes, with the well-established *E. coli* Nissle 1917, a probiotic strain with a notable history of application across various contexts. This comparative analysis aimed to gauge the relative efficacy of these two probiotics in conferring health benefits. The insights garnered from this comparison shed light on the underlying mechanisms that drive the beneficial properties of CEC15, underscoring the significance of such comparative investigations. While our current study primarily focused on assessing the specific characteristics of CEC15 that contribute to its probiotic properties, it is worth noting that there is room for further exploration in this field, involving comparing CEC15 with other *E. coli* strains, including commensal strains and those with established probiotic activities. Such studies could significantly enhance our understanding of the probiotic mechanisms at play. Additionally, investigating CEC15 in comparison to pathogenic strains holds the potential to elucidate the critical distinctions between fitness and pathogenicity within the *E. coli* strains. Although these avenues of analysis hold substantial importance, it's essential to clarify that they serve as prospects for future research, rather than the primary focus of our current study, which aimed to comprehensively evaluate the probiotic properties of CEC15. Below, we delve into what we consider the most pertinent aspects of these properties.

The first, which can be highlighted, is a genomic one. *E. coli*, a versatile bacterial species presents in the intestinal tract of many vertebrates, as well as in the external environment, is characterized by a great genetic, genomic, and phenotypic diversity among the strains it encompasses [46]. The *E. coli* species, which includes commensal and pathogenic strains, is divided into seven phylogroups, including four major phylogroups A, B1, B2, and D [47–49]. Whole genome phylogenetic analysis classified the two probiotic strains CEC15 and EcN into separate phylogroups. CEC15 clusters within the phylogroup B1, and EcN within B2. Among the *E. coli* phylogroups, the phylogroup B2 is the one most often associated with infections, especially urinary tract-related, and sepsis, followed by phylogroups A and D [50, 51], while members of the phylogroup B1 are more widely related to intestinal commensal bacteria of healthy animals [52]. In line with this result, CEC15 belongs to the O180:H14 serotype, which is mostly associated with non-pathogenic strains [53, 54], while the EcN serotype is O6:H1, a serotype often associated with pathogenic strains, especially enterotoxigenic *E. coli* (ETEC) and extraintestinal pathogenic *E. coli* (ExPEC) [55–57]. Although belonging to a phylogroup/serotype is not a safety indicator, it is nevertheless reassuring to note that CEC15 is phylogenetically close to commensal strains. Another important feature highly related to the phylogroup B2 is the presence of the *pks* island, allowing production of the genotoxic compound colibactin [58]. Auvray et al [59] isolated 785 *E. coli* strains from healthy bovines (*n* = 418), healthy humans (*n* = 278), and human sepsis (*n* = 89). Among those, 3%, 22%, and 39%, respectively, presented the *pks* island. On total, 42% of strains from the phylogroup B2 presented the *pks* island, while it was present in only 2% of strains from the phylogroup B1, from which none were isolated from human sepsis [59]. Interestingly, the CEC15 strain is devoid of *pks* island as well as of genes involved in colibactin synthesis. The *pks* island that was located on a chromosomal pathogenicity island 9 of strain EcN is present in various members of the *Enterobacteriaceae* family, particularly in *E. coli* and *Klebsiella pneumoniae* strains isolated from different sources, such as intestinal microbiota [60, 61], septicemia [62, 63], newborn meningitis [64], and urinary tract infections [65, 66]. These bacteria that produce colibactin are known to cause DNA damage and chromosomal instability in eukaryotic cells, leading to the senescence of epithelial cells and apoptosis of immune cells. Although many studies link the production of colibactin to the beneficial effect of the EcN strain, notably its anti-inflammatory effect [25, 26, 67–69], the absence of the *pks* gene cluster in strain CEC15 is an unambiguously advantageous feature exhibited by this promising probiotic.

Undesirable genetic traits such as virulence factors and antimicrobial resistances are often related to mobile genetic elements (MGEs) that can be acquired throughout adaptive evolution. The characterization of the mobilome of a probiotic strain, including phages, plasmids, genomic islands (GEIs), transposons, and insertion sequences (ISs), is therefore pivotal to evaluate its safety and to determine if its health-promoting benefits are acquired or intrinsic traits [70, 71]. Although GEIs were initially established in pathogenic bacteria, the comparison of DNA sequences from different microorganisms, including an increasing number of complete genome sequence of commensal and probiotic bacteria, has shown that regions with characteristics of GEIs can also be found in many non-pathogenic bacteria [72]. CEC15 is no exception. However, when compared to strain EcN, CEC15 presents a lower number of transposases and GEIs, including pathogenicity islands (PAI), metabolic islands (MI), and resistance islands (RI). Sequence analysis showed that, in general, a significant proportion of the gene clusters found in GEIs code for functions that aid in the survival and propagation of the strains. Hence, these genes may confer a selective advantage to microorganisms carrying the islands, when exposed to stress, *in vivo* conditions, or to antibacterial substances, by enhancing microbial transmission, survival, or colonization within a niche [73]. The lower GEIs content does not confer a disadvantage for CEC15 as the number of proteins linked to adaptation and survival on CEC15 genome is close to what is found on EcN genome.

Typically, the CEC15 PAI contain genes related to bacteria-bacteria competition, type II and IV secretion systems and the production of flagella and pili, while EcN PAI are composed mainly by type II and VI secretion systems, a wide variety of transposases, adhesion related genes and the *pks* gene cluster. In the context of a pathogen, all these features would represent a better chance for this pathogenic organism to begin a disease process. On the other hand, here in the context of two beneficial bacteria, these features could allow CEC15 and EcN to compete against pathogenic bacteria and to colonize the environment, leading to better chances to beneficially modulate the host response [74].

In the process of assessing the safety of strains, in addition to virulence factors, particular attention is given to the presence of antibiotic resistance determinants and their potential mobility [75]. Here, a total of 44 and 45 potential antimicrobial resistance (AMR) related genes were found on the EcN and CEC15 genome, respectively. These include genes coding for potential resistance to fluoroquinolones, β-lactams, macrolides, glycopeptides, and aminoglycosides. Antibiotic susceptibility testing (disc-diffusion method) was performed to confirm AMR gene prediction. This analysis has shown that besides having the larger number of AMR genes associated to fluoroquinolones and tetracycline, both strains were sensitive to the tested antibiotics from these antibiotic classes. Both strains displayed resistance to two lincosamides, lincomycin and clindamycin, and the macrolide erythromycin. This result corroborates genomic data with the presence of efflux pump genes such as *acrAB*-*tolC*, *emrAB*, *mdfA*, *emrE*, *acrE*, and *emrB*. In addition, both strains were resistant to oxacillin. However, neither strain presented the *bla* gene, associated with Extended-Spectrum β-Lactamase (ESBL). These enzymes can break down penicillin, cephalosporins (excluding cephamycin), and monobactams, but are not effective against carbapenems [76]. The *ampC* gene, on the other hand, was found on both strains and seems to be the responsible for Oxacilin resistance. Unlike ESBL, AmpC β-lactamase does not cause β-lactam resistance in wild strains [77, 78]. Finally, EcN was found resistant to kanamycin, while CEC15 was sensitive. Even if the presence of AMR genes is far from being wanted, AMR genes are detected into the genome of many commensal, food, and probiotic bacteria [79–84]. The presence of AMR genes is likely not a safety issue but can become when there is a risk of resistance transfer to other bacteria, notably to the human microbiota [75]. It has been proposed that if an AMR gene is found within 31 kb of an IS/transposon, it should be considered associated with the MGE, implying that it has the potential to be mobilized [85]. Among the AMR genes identified, few are at transposable distance from an IS in the genome of EcN (*n* = 6) and CEC15 (*n* = 12). Moreover, most of them are related to classes of antibiotics for which strains EcN and CEC15 are sensitive (fluoroquinolones and tetracycline). For genes likely involved in lincosamide and erythromycin resistance, only the EcN *tolC* and *ermE* genes could be transferable as close to an IS and within a GEI, respectively. This shows that, besides presenting a high number of AMR genes (44 in EcN and 45 in CEC15) a very low number is considered transferable, yet these genes are not the principal resistance gene related to a specific antibiotic class and, on CEC15, did not produce the phenotype.

Among the phenotypic different features identified between the two strains, the highest ability of strain CEC15 to tolerate acid and bile and to adhere to intestinal epithelial cells, two properties related to the survival and to the colonization of the human gastrointestinal tract (GIT), may be of great interest for a probiotic [45]. Indeed, *E. coli* has an impressive capability to endure low acidity levels and has various molecular mechanisms that facilitate this survival. The corresponding machinery can be expressed constantly, usually during a stationary phase, or triggered by different growth conditions [86]. We showed that strain CEC15 was more tolerant than strain EcN towards simulated gastrointestinal conditions and exhibited the highest survival rate during the intestinal phase. In the model here used, we applied a brutal change of pH from the initial to the gastric phase (pH 7 to pH 3) and then from the gastric to the intestinal phase (pH 3 to pH 7) whereas, *in vivo*, the pH would be much higher at the beginning of the gastric phase and then decrease slowly because of acidic secretions and gastric emptying. Therefore, the viability obtained with the INFOGEST model is probably underestimated. Similar differences in stress tolerance among *E. coli* strains were already reported in simulated human digestive environment [87]. Notably it has been shown that differences in acid resistance of strains were a consequence of their glutamate decarboxylase activity [86, 87]. Genomic comparison revealed that the genetic potentials associated to acid-resistance, including the decarboxylation of glutamate (*gadA*/*B*, *gadC*, *gadE*, *gadX*), were almost identical between CEC15 and EcN. Future work will be needed to determine whether the level of acid resistance of strains is linked to the production of the GAD system, its activity, or other mechanisms.

In addition to the survival under gastrointestinal conditions, mucosal adhesion is also a critical step for the establishment of probiotic strains in the gut, which is commonly viewed as a necessary requirement [88]. Numerous bacterial factors have been shown to be involved in adhesion to host surfaces [89]. Among the molecules involved in *E. coli* adhesion, flagella and pili/fimbriae are key actors during the initial attachment to surfaces [90]. As mentioned before, some genes associated to PAI are not exclusively associated to pathogenic traits. For instance, the possession of genes responsible for producing pili, which are frequently found inside PAI, gives the bacterium an advantageous position in various environments. Clusters of genes coding for pili were found in PAI 1 (type II secretion system for pseudopilin *gsp*) and PAI 13 (outer membrane usher protein *pef*) of CEC15 and were found on PAI 2 (fimbrial usher protein), PAI 3 (type II secretion system for pseudopilin *gsp*), PAI 4 (type II secretion system for pseudopilin *gsp*), and PAI 16 (Fimbrial S/F1C cluster) of EcN. From the 84 adhesins found on CEC15 genome, 34% were fimbriae/pili proteins while on EcN these proteins corresponded to 30% (of 89 proteins). Fimbriae/pili-like surface appendages are clearly seen on CEC15 electron microscopy images, by contrast with EcN images. As for the proteins detected on the surface of the strains, 14% of CEC15 exclusive proteins were fimbriae proteins while for EcN exclusive proteins they correspond to 5%.

The type 1 fimbriae of *E. coli*, especially the *fim* fimbriae gene cluster found exclusively on CEC15, have been demonstrated to facilitate the process of adhesion to epithelial host cells and contribute to the colonization of the intestinal tract [91]. Nonetheless, it is known that fimbriae/pili are hard to detect through proteomic analysis due to their structure and, consequently, resistance to proteolysis, especially by trypsin [92–94], which could be by-passed with the western-blot analysis of the different fimbriae types. The most frequent proteins detected belong to the F1C fimbriae family, associated to biofilm formation and intestinal strains by commensal strains, in special the EcN strain [95, 96]. According to Kleta et al., [95] F1C fimbriae, with H1 flagella also playing a role as bridges between EcN cells, as can be observed on the SEM images on Fig. 5, is the main protein responsible for adhesion capacities and the inhibitory effect against enteropathogenic *E. coli* (EPEC). The presence of these appendage-like proteins seen on the electron micrography and the detected proteins (notably type 1 fimbria chaperone FimC, type 1 fimbria D-mannose specific adhesin FimH, and type 1 fimbria minor subunit FimG and flagellin FliC) could correlate with the high adhesion of CEC15 to Caco-2 cells when compared to EcN. While the adhesion ability of probiotics to the host does not guarantee a health benefit, this interaction could lead to transient or permanent colonization, which may enhance their effects and hinder pathogen growth through competitive exclusion and bacterial antagonism mechanisms [97, 98]. Both a high survival rate, which could lead to many viable bacterial cells in the GIT, and a strong ability to attach to intestinal cells can be key factors enabling CEC15 to exert its probiotic activities *in vivo* and confer health benefits.

Bacterial components and metabolites of CEC15 and EcN were compared regarding their potential to modulate Caco-2 cells genetic expression. CEC15 and ECN modulated the gene expression of key factors for immunoregulation and epithelial integrity. In the conditions here tested, supernatant and inactivated bacteria, were able to promote some degree of modulation in most of the genes tested, notably the increased expression of Interleukin 8 (*Il8*). *Il8* has multiple effects on neutrophils, including their recruitment, activation of their granule release, induction of superoxide generation, and enhancement of adhesion molecule expression [99, 100]. It has been shown before that EcN is able to increase expression of *Il8* in different human intestinal epithelial cell lines, including Caco-2 cells, and that this increase is related mainly to EcN’s flagella [101], its capsule (K5) [102], and other unknown factors [103]. Both strains present similar *Il8* fold increase when stimulating with supernatant and inactivated cells at MOI 100 (~8-fold), where, yet, at low MOI only EcN was able to stimulate increased expression.

The Interleukin 1β (*Il1b*) gene expression was increased under CEC15 supernatant and inactivated bacteria at MOI 100 stimulation while no modulation was observed for the EcN strain. In healthy condition, the production of *Il1b* acts on the production of monocytes/macrophages, mediating innate immunity training, and promotes mucus secretion, induces proliferation and surface coagulability in barrier cells [104], essential activities to promote protection against pathogens.

CEC15 was able to modulate alone a few genes involved in host defense. The mucin 2 gene (*Muc2*) was stimulated by co-incubation with CEC15 at an MOI of 100. On the other hand, the expression of the Occludin gene (*Ocln*) was slightly reduced by stimulation with EcN co-incubation at MOI 100. Mucins are a crucial component of the intestinal barrier that protects against pathogens, and they form a major part of the intestinal mucous gel layer [105]. Our findings are consistent with those reported for other probiotic bacteria, such as *L. acidophilus* [106], *L. plantarum* [107], and *Lactobacillus* GG [108]. In addition, rats treated with the VSL#3 probiotic formula have been shown to exhibit an increase in colonic mucin secretion [109]. This agreement suggests that increased expression of *Muc2* may be a protective mechanism allowing probiotics to enhance intestinal barrier function and prevent pathogen colonization. In addition to its ability to antagonize pathogens, increased mucin production has been shown to enhance intestinal barrier function and provide protection against aggressions from luminal content or environmental matter [110].

The chemokine Monocyte chemoattractant protein-1 gene (*Mcp1*) was induced by all conditions of CEC15 while only EcN supernatant had similar effect. *Mcp1* is crucial in the regulation of septic shock as it facilitates the production of reactive oxygen species and various cytokines, which are vital components of the immune response against bacterial infections that can cause septic shock by attracting monocytes and other immune cells to the site of infection. [111]. At the same time, the expression of Tumor necrosis factor (*Tnf*) was induced by ECN in all conditions but only by CEC15 supernatant. In various inflammatory disorders, including Crohn's disease (CD), TNF-alpha is known to play a crucial role in intestinal inflammation and induce increase in the permeability of intestinal epithelial tight junctions (TJ). This increase in permeability can exacerbate the inflammatory response in the gut [112]. *Tnf* increased expression could be related to the decrease on expression of *Ocln* mentioned above. The Prostaglandin-endoperoxide synthase 2 (*Ptgs2*) was slightly stimulated by EcN, yet not by CEC15, which has been associated with the development of colorectal cancer [113, 114]. In summary, this result shows a more protective profile regarding CEC15 effects on increased expression of barrier genes and modulation of the immune system by increasing *Il1b* and *Mcp1* while EcN co-incubation led to increased pro-inflammatory genes.

Finally, *in vivo* studies were carried out to confirm the safety and effectiveness of CEC15 as a probiotic strain. For that, we administrate daily both strains to healthy mice, at high dosage (10^10^ CFU/mice/day) and evaluated their effects on the host and its intestinal microbiota after 13 days. To assess and compare their health effects, CEC15 and EcN strains were also tested in a mice 5-FU mucositis model. 5-FU is widely used as chemotherapeutic agent for the treatment of different types of cancer [115], it targets rapidly dividing and proliferating cells, effectively eradicating cancerous cells, and impeding their multiplication and division. Unfortunately, this process also affects healthy cells, especially those with a high proliferative rate, resulting in unwanted and detrimental side effects such as the intestinal mucositis [116, 117]. Therefore, chemotherapy impacts various aspects of the intestinal barrier, such as the mucus layer, epithelium, neuroendocrine feedback signaling, immune system, and gut vascular barrier [115]. These effects can lead to heightened immunological responses, increased intestinal permeability to toxins, and potentially facilitate the movement of bacteria from the gut into adjacent organs or the systemic circulation [118]. Given the critical role of the microbiota in sustaining a healthy gastrointestinal mucosa, a highly promising avenue for mitigating the adverse effects of 5-FU-induced mucositis could involve the utilization of probiotics, which hold significant potential in this regard [119]. In recent years, the significance of probiotics and their derivatives for the treatment of mucositis has been increasingly recognized [115]. This is underscored by the inclusion of probiotics in the guidelines of the Multinational Association of Supportive Care in Cancer and International Society for Oral Oncology (MASCC/ISOO) regarding mucositis management. The guidelines, which distill the most credible scientific evidence into practical clinical recommendations, indicate that probiotics containing *Lactobacillus* spp. could offer advantages in averting diarrhea induced by radiotherapy or chemotherapy in patients with pelvic malignancies [120]. Furthermore, in addition to the abovementioned, the reason for selecting this model is its high severity, which would enable the observation of notable protective effects promoted by any potential probiotic strains [7, 121, 122] and the fact that it is a well-established model [123–127]. Furthermore, it is crucial to emphasize the absence of studies assessing the impact of both EcN and CEC15 on the 5-FU-induced intestinal mucositis model, apart from two studies evaluating the effects of EcN supernatant [128, 129].

In healthy animals, with the criteria used, no detrimental effects were associated with the consumption of the two strains. We only observed a small reduction on crypt depth following the CEC15 administration, as previously observed when conventional and gnotobiotic rats colonized by CEC15 were compared [36]. Both strains were able to improve intestinal barrier and epithelial integrity as have been reported before [36, 37, 130–132]. Likewise, no significant variation in microbiota composition, richness and diversity was observed following probiotic administration, even though the microbiota of CEC15-treated mice seemed closer to that of control mice than those treated with strain EcN. As expected, 5-FU administration led to a consistent inflammatory process in the ileum, which was characterized by excessive weight loss, increase in intestinal permeability, neutrophils infiltration, and an accentuated destruction of ileal epithelial structure, as it has been reported by many studies before [123, 124, 127]. No strain was able to prevent weight loss, a result that is not surprising given the aggressive nature of the 5-FU therapy. Nevertheless, CEC15, yet not EcN, partially prevented the weight loss, and such protection is known to depend on the probiotic strain, as has been observed with many other probiotics [125, 127, 133–138]. While both strains prevented both the increase of intestinal permeability and the decrease of villus height, CEC15 intervention specifically and significantly reduced the histological score that reflects the architectural damages of tissues caused by 5FU treatment. It further prevented decrease of crypt depth and increase of MPO activity, a biomarker of inflammation and oxidative stress.

The 5-FU administration also resulted in an imbalance of the intestinal microbiota, as evidenced by a decreased abundance of *Firmicutes*, yet increased abundance of *Bacteroidota* and *Proteobacteria* in mice. Metagenomics studies conducted in both experimental animal models and patients with intestinal inflammatory diseases have reported conflicting results, with some studies showing a decrease in the *Firmicutes* phylum [139, 140]. Interestingly, CEC15 administration showed a large reduction on *Proteobacteria* restoring with no alteration in the *Firmicutes*/*Bacteroidota* ratio. As for EcN there was only a slight restoration with even an increase on *Bacteroidota*. Moreover, the treatment with CEC15 in 5-FU-induced mucositis promoted a degree of protection showing no difference to the NC group in relation to the diversity of the samples. Both *Firmicutes* and *Bacteroidota* phyla have been negatively correlated with mortality and DAI score [141], suggesting that restoring their abundance may have played an important role in the protection of the intestinal architecture. Altogether, our results suggest that, as EcN, CEC15 is also a strain to be safely administrated in healthy conditions.

In the context of this work, the *Parabacteroides* genus, which saw an increase in levels with EcN treatment, has garnered attention for its association with host health. Recent reports indicate its decrease in conditions such as inflammatory bowel disease and obesity, underscoring its significance. Furthermore, *Parabacteroides* exhibits physiological traits related to carbohydrate metabolism and the production of short-chain fatty acids [142]. Notably, *Parabacteroides* levels have risen in the EcN/MUC group in comparison to the control groups.

Conversely, several members of the *Eubacterium* genus are known for their butyrate production, playing pivotal roles in maintaining energy balance, regulating colonic motility, modulating the immune system, and suppressing inflammation within the gut [143]. *Eubacterium* species are also involved in transformations of bile acids and cholesterol, contributing to their regulation. Dysbiosis and altered representation of *Eubacterium* species have been linked to various human diseases [143]. Surprisingly, *Eubacterium* levels remained unaffected by 5-FU administration, and an increase was observed after treatment of healthy animals with CEC15, a phenomenon not observed with EcN. Additionally, the *Eubacterium* group, alongside the *Lachnospiraceae* NK4A136 group, are prolific producers of butyrate, a key compound involved in regulating gut inflammatory processes and immune system development [144]. Furthermore, the butyrate producing *Butyricicoccus* has displayed its ability to prevent necrotic enteritis and reduce pathogen abundance in the cecum and ileum [145]. The treatments with CEC15 and EcN led to increased levels of *Butyricoccus* compared to the control and mucositis-treated groups. Also, working in conjunction with *Lachnospiraceae*, *Lactobacillus* serves as a crucial group of bacteria responsible for synthesizing short-chain fatty acids [146]. Notably, CEC15 exhibited higher levels of *Lactobacillus* members than the EcN and MUC groups.

*Colidextribacter* demonstrates a significant correlation with inflammation-related serum metabolites from gut microbes, suggesting its potential role in producing inflammatory metabolites [147]. Interestingly, this genus was only elevated by EcN administration. Research has pointed to the *Clostridia* vadinBB60 group's inverse correlation with obesity, dyslipidemia, and insulin resistance in a mouse model [148–151]. Its reduction has also been associated with elevated trimethylamine N-oxide levels and an increased risk of thrombosis [148–151]. Notably, CEC15 treatment positively modulated this group. Furthermore, it has been reported that *Anaeroplasma* is strongly associated with intestinal IgA and TGF-B secretion, playing a key role in regulating intestinal inflammation [152]. In this study, only the CEC/MUC group exhibited positive modulation of the *Anaeroplasma* genus.

## Conclusions

All things considered, the commensal *E. coli* CEC15 has a potential as a probiotic strain, due to its ability to modulate the intestinal microbiota, provide protective and anti-inflammatory effects, and reinforce the intestinal barrier. The modulation of the microbiota, in especial by CEC15, has led to positive modulation of SCFA and anti-inflammatory-related genera. The study suggests that the CEC15 strain is effective against an inflammation model of 5FU-induced intestinal mucositis, which could translate to a treatment for patients under similar conditions. However, further research is needed to evaluate the safety and effectiveness of the CEC15 strain in humans.

## Methods

### In silico analysis

#### Strain, growth, and DNA extraction

Two *Escherichia coli* strains were used in this work. We previously isolated the primo-colonizing *E. coli* CEC15 (CEC15) strain from freshly pooled fecal samples of 15-day-old suckling rodents [36]. The probiotic *E. coli* Nissle 1917 (EcN) strain was kindly given by professor Flaviano Martins from Federal University of Minas Gerais, Brazil. For DNA extraction, CEC15 was grown on Luria-Bertani (LB) medium (1% peptone, 0.5% yeast extract, and 0.5% NaCl) for 24 h at 37 °C under shaking conditions (150 rpm). Colony Forming Units (CFUs) were enumerated by serial dilutions in peptone water prior to spreading on top of solid LB medium added with agar. DNA was extracted using Wizard® Genomic DNA Purification Kit (Promega, Wisconsin, EUA), according to the manufacturer’s instructions. DNA was quantified using the nanodrop 2000 spectrophotometer (ThermoFisher, Massachusetts, EUA) and proceeded to sequencing.

#### Genome sequencing, assembly, annotation, and phylogenomic analysis

DNA sequencing was performed using the Illumina HiSeq platform, with a pair-end library of 2x151 bp and an insert size of 450 bp (Göttingen, Germany), and by the PacBio platform. The analysis of the quality of the reads was performed using the software FastQC (FastQC: a quality control tool for high throughput sequence data. Available online at: http://www.bioinformatics.babraham.ac.uk/projects/fastqc). Data from Illumina sequencing generated a Phred value of 39 for 6280224*2; thus, trimming was unnecessary. Sequenced from PacBio presented 122,634 reads and, after the Phred value was adjusted to 24, a new file was generated with 12238 reads with a range size of 500-24781 bp.

The assembly was performed *ab initio* with the SPAdes software (v. 3.15.3 [Python version: 3.5.2]) [153] with a hybrid assembly approach from the two sequencing platforms’ results. A scaffold assembly was reached with 4,772,817 bp (four gaps) forming a chromosome, one contig of 201,163 bp representing the plasmid, and 26 contigs of 7588 bp also belonging to the chromosome; however, these 26 contigs were excluded from the analysis by an assembly quality filter for being recognized as an artifact of low sequencing coverage and without significative similarity by BLAST analysis to any *E. coli* genome deposited on NCBI database (April 6th, 2022). MOB-suite software [154] was used to classify contigs from the chromosome and the plasmid. The chromosome scaffold had its origin fixed at the *dnaA* gene, with a total of 5 gaps, and the plasmid had its origin fixed at the *repB* gene. The remaining gaps were closed using the software GFinisher (v. 1.4) [155] based on contig assembled by the software EDENA (v.3.131028) [156]. In the end, we have a chromosome with 4,780,804 bp, with sequence coverage of 383.49-fold and GC content of 50.78%, and a plasmid of 200,825 bp, with sequencing coverage of 604.24-fold and GC content of 47.25%. The software CLC Genomics Workbench (v. 22) was used for the final mapping of reads resulting in 99.71% of reads mapped. The sequence was deposited on NCBI under the access numbers CP133657.1 (chromosome) and CP133658.1 (pCEC15).

The genome of CEC15 and EcN strains (NCBI access: CP058217.1 [chromosome], CP058218.1 [pMUT1], and CP058219.1 [pMUT2]) used in this study were automatically annotated by the Prokaryotic Genome Annotation Pipeline (PGAP-NCBI) [157–159]. Functional annotation was performed with EggNOG-mapper [160, 161]. The orthology between the two genomes was analyzed by the OrthoFinder tool [162].

Twenty-two *E. coli* strains were subjected to phylogenomic analysis. We added 20 strains representing the *E. coli* phylogroups A, B1, B2, C, D, E, and F, to the CEC15 and EcN strains. The phylogenomic tree was constructed with the phylogenomic tree tool from PATRIC (https://www.patricbrc.org/app/PhylogeneticTree) by the codon tree method. In this method, the orthologous genes were identified via annotation of Protein Global Families (PGFams) of PATRIC [163]. The sequences of protein were aligned by MUSCLE software [164], and the corresponding codon sequences were concatenated. The phylogenomic inference was realized via the RAxML program [165] with support values estimated by 100 fast bootstrapping runs [166]. The tree was visualized and edited with the tool iTOL (v.6.53) (https://itol.embl.de/).

#### Genomic islands prediction, transposases, and insertion elements

Prediction of Metabolic (MI), Resistance (RI), and Pathogenicity (PAI) islands in CEC15 and EcN strains was performed with the software GIPSy (Genomic Island Prediction Software, v.1.1.3) [167], using *Escherichia coli* O157:H7 str. Sakai genome (NC_002695) as a reference. Phage islands were predicted utilizing PHASTER tool (PHAge Search Tool Enhanced Release) [168, 169]. Visualization of the genomic island’s map was performed with BRIG software (BLAST Ring Image Generator, v. 0.95) [170]. The annotation of insertion elements was done using the tool ISSaga (Insertion Sequence Semi-Automatic Genome Annotation) (http://issaga.biotoul.fr/) [39]. The serotyping of CEC15 was identified based on genes for specific O-antigen (O typing) and flagellin genes (H typing) with the SerotypeFinder 2.0 web tool hosted by the Center for Genomic Epidemiology (CGE) (www.genomicepidemiology.org). Data was curated manually and tabulated.

#### Antibiotic resistance genes

The identification of genes related to the resistance of antibiotic compounds in the genome of the CEC15 and EcN strains was performed by alignment to CARD (Comprehensive Antibiotic Resistance Database) [40], using the ABRicate (https://github.com/tseemann/abricate) software.

#### Bacteriocins, adhesin, stress response-related genes predictions, and metabolic profiling

Bacteriocins-coding genes were predicted with BAGEL4 (http://bagel4.molgenrug.nl/) [171]. The presence of adhesins proteins in the genomes of CEC15 and EcN was analyzed by SPAAN software (score>0.8) [44]. The identification of genes related to stress response (acid and osmolarity) was curated manually based on the protein function described on the UniProt database. Metabolic profiling was performed using the BlastKOALA tool (https://www.kegg.jp/blastkoala) [41].

### In vitro assays

#### Survival under simulated gastrointestinal conditions

CEC15 and EcN strains were grown on LB medium for 16 h at 37 °C under shaking conditions, the cultures were then diluted 100-fold in Simulated Gastric Fluid (SGF) (KCl 6.9 mM, K_2_HPO_4_ 0.9 mM, NaHCO_3_ 25 mM, NaCl 47.2 mM, MgCl_2_ 0.1 mM, (NH_4_)_2_CO_3_ 0.5 mM, and CaCl_2_ 0.15mM, pH 3) and submitted to the INFOGEST *in vitro* simulation of gastrointestinal food digestion [172] with some modifications. In brief, diluted cultures were centrifugated at 5000 x *g* for 10 min at 4 °C, the supernatant was removed, and the pellet was washed twice with sterile PBS prior to centrifugation. The washed pellet was then resuspended in 10 mL of SGF, at this point, an aliquot of 500 µL was collected for CFU counting (T1). To simulate the digestion, 200 U/mL of porcine pepsin (Sigma-Aldrich, cat. no. P7012) were added. SGF was added to a final volume of 20 mL, and the tubes were incubated in a water bath at 37 °C with agitation at 60 rpm for 2 h. After the incubation period, another 500 µL aliquot was collected for CFU counting (T2), and the samples passed to the intestinal phase where 20 mL of Simulated Intestinal Fluid (SIF) (KCl 6.8 mM, K_2_HPO_4_ 0.8 mM, NaHCO_3_ 85 mM, NaCl 38.4 mM, MgCl_2_ 0.33 mM, and CaCl_2_ 0.6 mM, pH 7) was added. The pH of the solution was adjusted to 7 using 1N NaOH and, to simulate the intestinal environment, 10 mM of bile salts (Sigma-Aldrich, cat. no. B3883) and pancreatin (equivalent to trypsin activity of 100 U/mL) (Sigma-Aldrich, cat. no. P7545) were added. The tubes were again incubated, as previously, for 2 h, and a final aliquot was collected for CFU counting (T3). CFU quantification was performed on LB agar plates, incubated at 37 °C overnight before manually counting colonies. The results were expressed in % of survival to the initial CFU. The experiment was done in triplicate.

#### Antibiotic susceptibility

The susceptibility towards antimicrobials was performed using the Kirby-Bauer method (disk diffusion). For that, 250 µL of overnight culture (CEC15 and EcN) on LB medium were placed in a Mueller-Hinton agar plate and spread evenly with the aid of a sterile swab, the plate was left open to dry for about 10 min, and four antibiotic disks were placed in each plate. The plates were then incubated at 37 °C for 20 h, and the halo was measured with a millimetric ruler. The following classes, and their respective antibiotics (BIO-RAD, France), were tested: Penicilins: Ampicillin (AMP, 10 µg), and Oxacillin (OXA, 5 µg); Quinolones: Ciprofloxacin (CIP, 5µg), Chloramphenicol (CHL, 30µg), Norfloxacin (NXN, 10µg), and Nalidixic acid (NAL, 30µg); Macrolides: Erythromycin (ERY, 15 µg); Aminoglycosides: Gentamicin (GMI, 15 µg), Kanamycin (KNM, 30 µg), Streptomycin (SMN, 10 µg) and Tobramycin (TMN 10µg); Tetracyclines: Tetracycline (TET, 30 µg); Lincosamides: Lincomycin (LCN, 15 µg) and Clindamycin (CMN, 2µg); Phosphonic antibiotics: Fosfomycin (FSF, 50 µg); Glycopeptides: Vancomycin (VAN, 30 µg); and Ansamycin antibiotics: Rifampicin (RAM, 30µg). The results were analyzed according to Clinical and Laboratory Standards Institute (CLSI) and the European Committee on Antimicrobial Susceptibility Testing (EUCAST) standards for *Enterobacteriaceae*, when available, and expressed as susceptible, intermediate, resistant, and Area of Technical Uncertainty (ATU).

#### Hemolytic activity assay

For this assay, bacteria (CEC15 and EcN) were grown on LB medium overnight, and 10 µL of each culture were spotted in blood agar, supplemented with defibrinized sheep blood (5%), and incubated overnight at 37 °C. The strains *Staphylococcus aureus* BK and IT2 were used as α - and β-hemolytic strain control, respectively. The *S. aureus* was grown in BHI broth at 37 °C and 150 rpm overnight and 10 µL was spotted on the plate as described for *E. coli* strains. The results are expressed as α-hemolysis (presence of a greenish halo around the bacteria), β-hemolysis (presence of a clear halo), and γ-hemolysis (no halo).

#### Adhesion assay in human colon carcinoma (Caco-2) cells

The human Caco-2 colon adenocarcinoma cell line (ATCC-HTB-37) was cultured in DMEM high glucose (DMEM-HG) medium supplemented with 10% (v/v) fetal bovine serum (FBS),100 U/mL penicillin and 100 µg/mL streptomycin (P0781, Sigma-Aldrich®). The cells were seeded in a 75 cm^2^ flask at a density of 1x10^4^ cells/cm^2^ and incubated at 37 °C and 5% CO_2_ until reached 80% confluence. The cells were washed twice with phosphate-buffered saline (PBS) and detached with trypsin 0.25% for 5 min at 37 °C. Live cells were counted using the TC20 Automated Cell Counter (BIO-RAD) with trypan blue staining. A 12-well plate was prepared by seeding 7x10^4^ cells/well, and it was kept at 37 °C and 5% CO_2_ for 21 days until differentiation. The culture medium was changed every 2 to 3 days for flasks and plates.

After 21 days of differentiation, the adhesion assay proceeded. For this, an overnight culture of *E. coli* (CEC15 and EcN) was diluted to 1% in fresh LB broth and incubated until reaching an optical density (OD_600nm_) of 0.5 (≈ 2 x10^8^ CFU/mL). One mL sample of bacterial cultures was centrifuged at 6000 x *g* for 10 min, and the pellet resuspended in 1 mL of DMEM-HG medium without FBS, penicillin and streptomycin. One aliquot was collected to calculate the initial CFU. The Caco-2 monolayers were washed with PBS and incubated with DMEM-HG medium containing 2 x10^8^ bacterial cells corresponding to a multiplicity of infection of 100 bacteria for each Caco-2 cell (MOI 100). After 2 h of incubation, the monolayers were then washed extensively three times with PBS to remove unattached bacteria. Caco-2 cells and adherent bacteria were then detached by the addition of 0.5 mL of trypsin (0.25%) and incubated for 5-7 min. Trypsin was neutralized by adding 0.5 mL of DMEM-HG with FBS. The cell suspension was then centrifuged at 6000 x* g* for 10 min at 4 °C, and the pellet was resuspended in 1 mL of Triton 0.1% in water to detach bacteria from Caco-2 cells. Serial dilutions of the cells suspension were plated on LB agar and incubated overnight for counting of viable bacteria. Adhesion experiments were performed in triplicates and expressed as % of adhered bacteria to Caco-2 cells in relation to the initial bacterial CFU added.

#### Scanning and Transmission Electron Microscopy (SEM and TEM)

For scanning electron microscopy (SEM) observations, 16-hours-old CEC15 and EcN cultures in LB were filtrated through 0.22 µm pore size nitrocellulose filter membrane, which were then cut into small pieces and placed into a fixation solution (2.5% glutaraldehyde, 100 mM sodium cacodylate). After 24 h, the filter pieces were transferred to a fresh solution of 0.25% glutaraldehyde and 100 mM sodium cacodylate. For SEM observations, the filters were removed from fixating solution, washed with fresh solution (0.25% glutaraldehyde and 100mM sodium cacodylate), dehydrated with ethanol (10, 25, 50, 75, 95, and finally 100%), CO_2_ dried, and coated with gold. The filter membranes were examined and photographed with a JEOL JSM-7100F scanning electron microscope, operating at 10 kV.

For transmission electron microscopy (TEM) observations, 16-hours-old CEC15 and EcN cultures were centrifugated (5,000 x *g*, 5 min), and the bacterial pellets were resuspended in the above fixation solution. After 24 h, the fixation solution was removed, and the bacterial pellets resuspended in 0.25% glutaraldehyde and 100 mM sodium cacodylate. The pellets were post-fixed with 1% osmium tetroxide containing 1.5% potassium cyanoferrate and 2% uranyl acetate in water before gradual dehydration in ethanol (30% to 100%) and embedding in Epon resin. Thin sections (70 nm) were collected on 200-mesh copper grids and counterstained with lead citrate before the examination. Fresh non-fixated samples were also examined by TEM, where a glow-discharged formvar-coated copper EM grid was placed on a drop of bacterial culture for 1 min, blotted with a filter paper, placed on a drop of 2% uranyl acetate for 1 min, blotted again, and air dried. All samples (fixed and fresh) were analyzed with JEOL 1400 transmission electron microscope (JEOL Ltd.) operating at 120 kV.

#### Shearing of fimbriae proteins

Overnight still-grown cultures (37 °C and no agitation) were centrifugated at 10000 x *g* for 10 min and the harvested cells were resuspended in PBS at 1/100 the initial culture volume. Fimbriae proteins were sheared using a waring blender at maximum speed for 5 min, two aliquots were collected, before and after shearing, and centrifuged at 10000 x *g* for 30 min to remove cells and debris. The resulting supernatant was collected, the protein content was quantified and resolved on precast NuPAGE Bis-Tris gradient gels (4-12%, ThermoFisher Scientific) for profile verification.

#### Proteomic analysis

Three independent replicates of shearing-derived proteins (10 µg each) were separated on 12% home-made SDS-PAGE minigels (Miniprotean II, Bio-Rad) and stained with Coomassie-blue (BIO-RAD, France). In-gel trypsin digestion was performed as described before [173]. Peptides were identified by mass spectrometry as described elsewhere [174], followed by protein identification (maximum e-value of 0.05) from the MS/MS spectra with the X!TandemPipeline software [175]. The peptides were searched against the genome sequences of the two strains described above with parameters as described before [176]. A minimum of 3 peptides per protein was necessary for the validation of the identification and a protein was only considered present when it was identified in at least two of the three replicates. The relative quantification of proteins was obtained by the Exponentially Modified Protein Abundance Index (emPAI) [177]. Proteins were categorized into Clusters of Orthologous Groups (COG).

#### Modulation of Caco-2 cells

Sixteen-hours-old CEC15 and EcN cultures in LB were diluted 10 and 100-fold and inactivated by heating at 60 °C for 1 hour. Inactivated cultures were centrifuged (5000 x *g*, 10 min) and the bacterial pellets were resuspended in 1 mL of DMEM-HG with FBS and antibiotics. The bacterial culture supernatants were prepared as follows. 1 mL of 16-hours-old CEC15 or EcN culture was centrifuged as described above, and the supernatant was filtered (0.22 µm pore diameter). Caco-2 cells were prepared as described above. For this assay, 6-well plates were prepared by seeding 1x10^5^ cells/well and incubated at 37 °C and 5% CO_2_ for 21 days until differentiation. The media was changed every 2 to 3 days. On the day of the assay, the medium was removed, and cells were washed twice with sterile PBS. The PBS was then replaced by DMEM (control), DMEM containing inactivated bacteria at MOI 10 and MOI 100, DMEM + EVs at the concentration of 1x10^9^ and 1x10^10^ EVs/mL, and DMEM with bacterial culture supernatant (final dilution of 100-fold). The plate was incubated for 24 h at 37 °C and 5% CO_2_. After incubation the supernatant was removed, and the cells were washed with PBS to remove the media and bacteria. The assay was performed in three independent experiments.

#### RT-qPCR assay

Total RNA was extracted using the RNeasy mini kit (Qiagen, Hilden, Germany) according to the manufacturer’s instructions. The cDNA was prepared from 1 µg of RNA using the qScript cDNA Synthesis Kit (Quantabio - Beverly, MA, EUA). The qPCR analysis was performed using the iQ™ SYBR® Green Supermix (BIO-RAD - Hercules, California, EUA) according to the manufacturer for a final volume of 20 µL and run in the CFX96 Real-Time system Thermal cycler (BIO-RAD - Hercules, California, EUA) with the following program: 95 °C for 3 min, 40 cycles of 95 °C for 10 s and 60 °C for 30 s, followed by a melting curve 55 °C – 95 °C increasing 0.5 °C per cycle. Data were analyzed by the 2^-ΔΔ^CT method for the reference genes (*GAPDH*, *b2m*, and H*prt1*). The list of primers used can be found in Additional file 18.

### In vivo assays

#### Experimental design

Male BALB/c mice, 4-5 weeks old with specific pathogen-free (SPF) status were obtained from the “Biotério central” of the Federal University of Minas Gerais (UFMG). Mice were randomly divided into 6 groups (8 animals per group) and kept in a microisolator (*n* = 4 each) with a 12 h light/dark cycle, temperature of 25 °C ± 2, and sterile filtrated water and standard chow food *ad libitum*. The experiment was conducted in agreement with the Brazilian College of Animal Experimentation (COBEA) and approved by the Use of Animals Ethics Committee from UFMG (CEUA – UFMG) under the protocol 67/2021.

For 12 days, mice were gavaged with 300 µL of sterile PBS (negative control group [NC] and mucositis group [MUC]), of *E. coli* CEC15 (1x10^10^ CFU) (CEC15 control group [CEC15] and CEC15 treatment group [CEC15-MUC]), or *E. coli* Nissle 1917 (1x10^10^ CFU) (EcN control group [EcN] and EcN treatment group [EcN-MUC]). On the 10^th^ day of experiments, the animals from the groups MUC, CEC15-MUC, and EcN-MUC received an intraperitoneal injection of 5-fluorouracil (5-FU, 300 mg/kg) to induce intestinal mucositis, while the other groups received injection of sterile PBS. On the last day of experimentation, to evaluate the intestinal permeability, all mice received by gavage 100 µL of a solution containing 18.5 MBq of diethylenetriamine penta-acetic acid labeled with technetium-99m (99mTc-DTPA) showing radiochemical purity of 99.4% performed by chromatography on Wattman paper. After 4 h, all mice were euthanized by anesthetic deepening (300 mg/mL of ketamine and 30 mg/mL of xylazine) (Ceva, São Paulo, Brazil), the blood was collected for permeability assay, and the ileum was collected for the remaining analyses. Water and food consumption, as well as animal weight, were evaluated daily for the duration of the experiment.

#### Permeability analysis

The blood was weighed and placed in appropriate tubes to determine radioactivity levels using an automated gamma counter (PerkinElmer Wallac Wizard 1470–020 Gamma Counter; PerkinElmer Inc., Waltham, EUA). The results are presented as the percentage of the radiation dose, which was calculated by the % dose per gram of ^99m^Tc-DTPA in blood following the equation:

$$\%dose/g= \frac{cpm \,in \,gram \,of \,blood}{cpm \,of \,standard} \times 100$$

were cpm = counts (of radioactivity) per minute.

#### Histopathological analysis

A section of approximately 4 cm of ileum was opened, washed with PBS to remove fecal matter, rolled up, and fixated with a 10% formalin solution. Later, tissue was embedded in paraffin, and sections of 4 µm were placed in microscope slides and stained with hematoxylin and Eosin (HE).

From each animal, 10 pictures from different tissue sections were collected using a BX41 optical microscope (Olympus, Tokyo, Japan) (20x). The pictures were blindly scored according to the system previously described by Howarth et al. [178], and the villus height and crypt depth (20 per animal) were measured with the assistance of the Image-J software (v. 1.51j.8 – NIH, Bethesda, MD, USA).

#### Neutrophilic infiltration assay

Neutrophilic infiltration was evaluated by detecting the myeloperoxidase enzyme activity (MPO assay) as described elsewhere [125]. Briefly, 50 mg of ileum were homogenized by maceration, centrifugated, and lysed by hypotonic solution, followed by three cycles of freezing in liquid nitrogen. After the last thawing samples were centrifugated and the supernatant was used for MPO assay (colorimetric). The assay absorbance was read at 450 nm and the results were expressed as MPO arbitrary units/ mg of tissue.

#### 16S rRNA amplicon metagenome analysis

Total DNA was extracted from fresh pooled feces of mice collected on the day of the euthanasia. An average of 50 mg of feces was used and the DNA extraction was performed with the QIAamp DNA stool Mini kit (QIAGEN) following the manufacture’s instruction. Library preparation and sequencing were performed as described before [124].

The FASTQ files underwent quality filtering, involving the removal of truncated and low-quality reads (those with a Phred score < 20), which was carried out using Trimmomatic [179]. Subsequently, the forward and reverse paired reads were merged to form contigs. These sequences were then subjected to a series of processing steps, which included dereplication, sorting by abundance, removal of singletons, and filtering for chimeric sequences using mothur [180]. Following this preprocessing, the sequences were clustered into Operational Taxonomic Units (OTU) at a 97% similarity threshold and taxonomically assigned using QIIME2 [181], with the taxonomic assignments being based on a 97% sequence similarity to the SILVA database [182].

### Statistical analysis

All *in vitro* experiments were done in triplicate while the *in vivo* experiments were performed with a technical duplicate. The results are presented as the mean ± the standard deviation. The *in vitro* and *in vivo* analysis were submitted to ANOVA test followed by the post-test of *Tukey*. The data of relative abundance of OTU were analyzed using ANOVA followed by Bonferroni multiple comparison test. The graphics were plotted on GraphPad Prism 7.0 where a *p*-value under 0.05 was statistically significant.

### Supplementary Information

**Additional file 1: Supplementary Table S1.** Genomic Islands identified in the Escherichia coli strain CEC15 genome by Gipsy software.

**Additional file 2: Supplementary Table S2.** Prophages regions identified in the Escherichia coli strain CEC15 genome by PHASTER tool.

**Additional file 3: Supplementary Table S3.** Genomic Islands identified in the Escherichia coli strain Nissle 1917 genome by Gipsy software

**Additional file 4: Supplementary Table S4.** Prophages regions identified in the Escherichia coli strain Nissle1917 genome by PHASTER tool.

**Additional file 5: Supplementary Table S5.** Putative insertion sequence family predicted in Escherichia coli CEC15 genome using ISSaga tool.

**Additional file 6: Supplementary Table S6.** Putative insertion sequence family predicted in Escherichia coli EcN genome using ISSaga tool.

**Additional file 7: Supplementary Figure S1.** Schematic circular representation of CEC15 genomic features.

**Additional file 8: Supplementary Figure S2.** Schematic circular representation of EcN genomic features.

**Additional file 9: Supplementaty Table S7.** Genes involved in resistance to antibiotic mechanisms in Escherichia coli CEC15.

**Additional file 10: Supplementary Table S8.** Genes involved in resistance to antibiotic mechanisms in Escherichia coli Nissle 1917.

**Additional file 11: Supplementary Table S9.** Metabolic profilling

**Additional file 12: Supplementary Table S10.** Proteins of Escherichia coli CEC15 and EcN with high adhesin profile probability

**Additional file 13: Supplementary figure S3.** Pre- and post-shearing protein profile of CEC15 and EcN strains.

**Additional file 14: Supplementary Table S11.** Proteins identification of pre-shearing and post-shearing samples of E. coli CEC15 and E. coli Nissle 1917

**Additional file 15: Supplementary figure S4.** Bacteriocins-encoding genes present in the genome of CEC15 and EcN strains.

**Additional file 16: Supplementary figure S5.** Relative gene expression of Caco-2 cells is unaltered by treated with either CEC15 or EcN strains

**Additional file 17: Supplementary table S12.** Number of OTUs per sample at the genus level.

**Additional file 18: Supplementary Table S13.** Primers used in this study

## Data Availability

The datasets generated and/or analyzed during the current study are available in the figshare repository, http://doi.org/10.6084/m9.figshare.23657679. The genome sequence of CEC15 was deposited on NCBI under the access numbers CP133657.1 (chromosome) and CP133658.1 (plasmid - pCEC15). Other data supporting the conclusions of this article are included in this published article [and its supplementary information files].

## References

[1] Stadlbauer V (2015). Immunosuppression and probiotics: are they effective and safe?. Benef Microbes.

[2] Hill C, Guarner F, Reid G, Gibson GR, Merenstein DJ, Pot B (2014). Expert consensus document. The International Scientific Association for Probiotics and Prebiotics consensus statement on the scope and appropriate use of the term probiotic. Nat Rev Gastroenterol Hepatol.

[3] Rijkers GT, de Vos WM, Brummer RJ, Morelli L, Corthier G, Marteau P (2011). Health benefits and health claims of probiotics: bridging science and marketing. Br J Nutr.

[4] Sniffen JC, McFarland LV, Evans CT, Goldstein EJC (2018). Choosing an appropriate probiotic product for your patient: an evidence-based practical guide. PLoS One.

[5] Jankiewicz M, Łukasik J, Kotowska M, Kołodziej M, Szajewska H (2023). Strain-specificity of Probiotics in Pediatrics: a Rapid Review of the clinical evidence. J Pediatr Gastroenterol Nutr.

[6] Bron PA, Tomita S, Mercenier A, Kleerebezem M (2013). Cell surface-associated compounds of probiotic lactobacilli sustain the strain-specificity dogma. Curr Opin Microbiol.

[7] Batista VL, da Silva TF, de Jesus LCL, Coelho-Rocha ND, Barroso FAL, Tavares LM (2020). Probiotics, Prebiotics, Synbiotics, and Paraprobiotics as a Therapeutic Alternative for Intestinal Mucositis. Front Microbiol.

[8] Zhao Z, Xu S, Zhang W, Wu D, Yang G (2022). Probiotic Escherichia coli NISSLE 1917 for inflammatory bowel Disease applications. Food Funct.

[9] Nißle A (1916). Ueber die Grundlagen Einer Neuen ursächlichen Bekämpfung Der Pathologischen Darmflora. DMW - Deutsche Medizinische Wochenschrift.

[10] Wassenaar TM (2016). Insights from 100 years of Research with Probiotic *E. Coli*. Eur J Microbiol Immunol (Bp).

[11] Nißle A (1918). Die antagonistische Behandlung Chronischer Darmstörungen Mit Colibakterien. Med Klin.

[12] Sonnenborn U (2016). Escherichia coli strain Nissle 1917—from bench to bedside and back: history of a special *Escherichia coli* strain with probiotic properties. FEMS Microbiol Lett.

[13] Mohsin M, Guenther S, Schierack P, Tedin K, Wieler LH (2015). Probiotic Escherichia coli Nissle 1917 reduces growth, Shiga toxin expression, release and thus cytotoxicity of enterohemorrhagic Escherichia coli. Int J Med Microbiol.

[14] Pradhan S, Weiss AA (2020). Probiotic properties of Escherichia coli Nissle in Human Intestinal Organoids. mBio.

[15] Behrouzi A, Mazaheri H, Falsafi S, Tavassol ZH, Moshiri A, Siadat SD (2020). Intestinal effect of the probiotic Escherichia coli strain Nissle 1917 and its OMV. J Diabetes Metab Disord.

[16] Hare PJ, Englander HE, Mok WWK (2022). Probiotic Escherichia coli Nissle 1917 inhibits bacterial persisters that survive fluoroquinolone treatment. J Appl Microbiol.

[17] Olbertz D, Proquitté H, Patzer L, Erler T, Mikolajczak A, Sadowska-Krawczenko I (2022). Potential Benefit of Probiotic E. Coli Nissle in Term neonates. Klin Padiatr.

[18] Teng G, Liu Z, Liu Y, Wu T, Dai Y, Wang H (2022). Probiotic Escherichia coli Nissle 1917 expressing elafin protects against inflammation and restores the gut microbiota. Front Microbiol.

[19] Chiang CJ, Chao YP, Ali A, Day CH, Ho TJ, Wang PN (2021). Probiotic *Escherichia coli* Nissle inhibits IL-6 and MAPK-mediated cardiac hypertrophy during STZ-induced Diabetes in rats. Benef Microbes.

[20] Faghihi AH, Agah S, Masoudi M, Ghafoori SMS, Eshraghi A (2015). Efficacy of Probiotic Escherichia coli Nissle 1917 in patients with irritable bowel syndrome: a double blind placebo-controlled Randomized Trial. Acta Med Indones.

[21] Cress BF, Linhardt RJ, Koffas MAG (2013). Draft Genome Sequence of Escherichia coli Strain Nissle 1917 (Serovar O6:K5:H1). Genome Announc.

[22] Reister M, Hoffmeier K, Krezdorn N, Rotter B, Liang C, Rund S (2014). Complete genome sequence of the Gram-negative probiotic Escherichia coli strain Nissle 1917. J Biotechnol.

[23] Homburg S, Oswald E, Hacker J, Dobrindt U (2007). Expression analysis of the colibactin gene cluster coding for a novel polyketide in *Escherichia coli*. FEMS Microbiol Lett.

[24] Morgan RN, Saleh SE, Farrag HA, Aboulwafa MM (2019). Prevalence and pathologic effects of colibactin and cytotoxic necrotizing factor-1 (cnf 1) in Escherichia coli: experimental and bioinformatics analyses. Gut Pathog.

[25] Olier M, Marcq I, Salvador-Cartier C, Secher T, Dobrindt U, Boury M (2012). Genotoxicity of Escherichia coli Nissle 1917 strain cannot be dissociated from its probiotic activity. Gut Microbes.

[26] Massip C, Branchu P, Bossuet-Greif N, Chagneau CV, Gaillard D, Martin P (2019). Deciphering the interplay between the genotoxic and probiotic activities of Escherichia coli Nissle 1917. PLoS Pathog.

[27] Krumbeck JA, Rasmussen HE, Hutkins RW, Clarke J, Shawron K, Keshavarzian A (2018). Probiotic Bifidobacterium strains and galactooligosaccharides improve intestinal barrier function in obese adults but show no synergism when used together as synbiotics. Microbiome.

[28] Forsythe P, Bienenstock J (2010). Immunomodulation by commensal and probiotic Bacteria. Immunol Invest.

[29] Anjana, Tiwari SK. Bacteriocin-Producing Probiotic Lactic Acid Bacteria in Controlling Dysbiosis of the Gut Microbiota. Front Cell Infect Microbiol. 2022;12:851140. 10.3389/fcimb.2022.851140 PMC9149203 35651753

[30] Mills JP, Rao K, Young VB (2018). Probiotics for prevention of Clostridium difficile Infection. Curr Opin Gastroenterol.

[31] Ventura M, O’Flaherty S, Claesson MJ, Turroni F, Klaenhammer TR, van Sinderen D (2009). Genome-scale analyses of health-promoting bacteria: probiogenomics. Nat Rev Microbiol.

[32] de Melo Pereira GV, de Oliveira Coelho B, Magalhães Júnior AI, Thomaz-Soccol V, Soccol CR (2018). How to select a probiotic? A review and update of methods and criteria. Biotechnol Adv.

[33] Castro-López C, García HS, Guadalupe Martínez-Ávila GC, González-Córdova AF, Vallejo-Cordoba B, Hernández-Mendoza A (2021). Genomics-based approaches to identify and predict the health-promoting and safety activities of promising probiotic strains – A probiogenomics review. Trends Food Sci Technol.

[34] Ventura M, Turroni F, van Sinderen D (2012). Probiogenomics as a tool to obtain genetic insights into adaptation of probiotic bacteria to the human gut. Bioengineered.

[35] Carvalho RDO, Guédon E, Aburjaile FF, Azevedo V (2022). Editorial: Probiogenomics of classic and next-generation probiotics. Front Microbiol.

[36] Tomas J, Reygner J, Mayeur C, Ducroc R, Bouet S, Bridonneau C (2015). Early colonizing Escherichia coli elicits remodeling of rat colonic epithelium shifting toward a new homeostatic state. ISME J.

[37] Escribano-Vazquez U, Verstraeten S, Martin R, Chain F, Langella P, Thomas M (2019). The commensal Escherichia coli CEC15 reinforces intestinal defences in gnotobiotic mice and is protective in a chronic Colitis mouse model. Sci Rep.

[38] Bay DC, Rommens KL, Turner RJ (2008). Small multidrug resistance proteins: a multidrug transporter family that continues to grow. Biochim Biophys Acta.

[39] Varani AM, Siguier P, Gourbeyre E, Charneau V, Chandler M (2011). ISsaga is an ensemble of web-based methods for high throughput identification and semi-automatic annotation of insertion sequences in prokaryotic genomes. Genome Biol.

[40] Alcock BP, Raphenya AR, Lau TTY, Tsang KK, Bouchard M, Edalatmand A (2019). CARD 2020: antibiotic resistome surveillance with the comprehensive antibiotic resistance database. Nucleic Acids Res.

[41] Kanehisa M, Sato Y, Morishima K (2016). BlastKOALA and GhostKOALA: KEGG Tools for functional characterization of genome and metagenome sequences. J Mol Biol.

[42] Castanié-Cornet M-P, Cam K, Bastiat B, Cros A, Bordes P, Gutierrez C (2010). Acid stress response in Escherichia coli: mechanism of regulation of gadA transcription by RcsB and GadE. Nucleic Acids Res.

[43] Battesti A, Majdalani N, Gottesman S (2011). The RpoS-Mediated general stress response in *Escherichia coli*. Annu Rev Microbiol.

[44] Sachdeva G, Kumar K, Jain P, Ramachandran S (2005). SPAAN: a software program for prediction of adhesins and adhesin-like proteins using neural networks. Bioinformatics.

[45] Shokryazdan P, Faseleh Jahromi M, Liang JB, Ho YW (2017). Probiotics: from isolation to application. J Am Coll Nutr.

[46] Blount ZD (2015). The unexhausted potential of E. coli. Elife.

[47] Clermont O, Gordon D, Denamur E (2015). Guide to the various phylogenetic classification schemes for Escherichia coli and the correspondence among schemes. Microbiol (N Y).

[48] Clermont O, Dixit OVA, Vangchhia B, Condamine B, Dion S, Bridier-Nahmias A (2019). Characterization and rapid identification of phylogroup G in *Escherichia coli*, a lineage with high virulence and antibiotic resistance potential. Environ Microbiol.

[49] Waters NR, Abram F, Brennan F, Holmes A, Pritchard L (2020). Easy phylotyping of Escherichia coli via the EzClermont web app and command-line tool. Access Microbiol.

[50] Jauréguy F, Carbonnelle E, Bonacorsi S, Clec’h C, Casassus P, Bingen E (2007). Host and bacterial determinants of initial severity and outcome of Escherichia coli Sepsis. Clin Microbiol Infect.

[51] Jaureguy F, Landraud L, Passet V, Diancourt L, Frapy E, Guigon G (2008). Phylogenetic and genomic diversity of human bacteremic Escherichia coli strains. BMC Genomics.

[52] Sarowska J, Olszak T, Jama-Kmiecik A, Frej-Madrzak M, Futoma-Koloch B, Gawel A (2022). Comparative characteristics and pathogenic potential of Escherichia coli isolates originating from Poultry farms, Retail Meat, and human urinary tract Infection. Life.

[53] González-Escalona N, Kase JA (2019). Virulence gene profiles and phylogeny of Shiga toxin-positive Escherichia coli strains isolated from FDA regulated foods during 2010–2017. PLoS ONE.

[54] Fabian NJ, Mannion AJ, Feng Y, Madden CM, Fox JG (2020). Intestinal colonization of genotoxic Escherichia coli strains encoding colibactin and cytotoxic necrotizing factor in small mammal pets. Vet Microbiol.

[55] Johnson JR, Johnston B, Clabots CR, Kuskowski MA, Roberts E, DebRoy C (2008). Virulence genotypes and phylogenetic background of Escherichia coli Serogroup O6 isolates from humans, dogs, and cats. J Clin Microbiol.

[56] Beutin L, Delannoy S, Fach P (2016). Genetic Analysis and detection of fliCH1 and fliCH12 genes coding for Serologically closely related Flagellar antigens in Human and Animal Pathogenic Escherichia coli. Front Microbiol.

[57] Pacheco ABF, Guth BEC, Soares KCC, Almeida DF, Ferreira LCS (2006). Clonal relationships among Escherichia coli serogroup O6 isolates based on RAPD. FEMS Microbiol Lett.

[58] Faïs T, Delmas J, Barnich N, Bonnet R, Dalmasso G (2018). Colibactin: more than a New Bacterial Toxin. Toxins (Basel).

[59] Auvray F, Perrat A, Arimizu Y, Chagneau CV, Bossuet-Greif N, Massip C (2021). Insights into the acquisition of the pks island and production of colibactin in the Escherichia coli population. Microb Genom.

[60] Dubois D, Delmas J, Cady A, Robin F, Sivignon A, Oswald E (2010). Cyclomodulins in Urosepsis strains of Escherichia coli. J Clin Microbiol.

[61] Putze J, Hennequin C, Nougayrède J-P, Zhang W, Homburg S, Karch H (2009). Genetic structure and distribution of the Colibactin Genomic Island among members of the Family *Enterobacteriaceae*. Infect Immun.

[62] Johnson JR, Johnston B, Kuskowski MA, Nougayrede J-P, Oswald E (2008). Molecular epidemiology and phylogenetic distribution of the Escherichia coli pks genomic island. J Clin Microbiol.

[63] Nowrouzian FL, Oswald E (2012). Escherichia coli strains with the capacity for long-term persistence in the bowel microbiota carry the potentially genotoxic pks island. Microb Pathog.

[64] McCarthy AJ, Martin P, Cloup E, Stabler RA, Oswald E, Taylor PW (2015). The Genotoxin Colibactin is a determinant of virulence in Escherichia coli K1 experimental neonatal Systemic Infection. Infect Immun.

[65] Krieger JN, Dobrindt U, Riley DE, Oswald E (2011). Acute Escherichia coli Prostatitis in previously Health Young men: bacterial virulence factors, Antimicrobial Resistance, and clinical outcomes. Urology.

[66] Micenková L, Beňová A, Frankovičová L, Bosák J, Vrba M, Ševčíková A (2017). Human Escherichia coli isolates from hemocultures: Septicemia linked to urogenital tract Infections is caused by isolates harboring more virulence genes than bacteraemia linked to other conditions. Int J Med Microbiol.

[67] Reuter C, Alzheimer M, Walles H, Oelschlaeger TA (2018). An adherent mucus layer attenuates the genotoxic effect of colibactin. Cell Microbiol.

[68] Li R, Helbig L, Fu J, Bian X, Herrmann J, Baumann M (2019). Expressing cytotoxic compounds in Escherichia coli Nissle 1917 for tumor-targeting therapy. Res Microbiol.

[69] Bian X, Plaza A, Zhang Y, Müller R (2015). Two more pieces of the colibactin genotoxin puzzle from Escherichia coli show incorporation of an unusual 1-aminocyclopropanecarboxylic acid moiety. Chem Sci.

[70] Leplae R, Lima-Mendez G, Toussaint A (2010). ACLAME: a CLAssification of Mobile genetic elements, update 2010. Nucleic Acids Res.

[71] Mahillon J, Chandler M (1998). Insertion sequences. Microbiol Mol Biol Rev.

[72] Dobrindt U, Hochhut B, Hentschel U, Hacker J (2004). Genomic islands in pathogenic and environmental microorganisms. Nat Rev Microbiol.

[73] Hacker J, Carniel E (2001). Ecological fitness, genomic islands and bacterial pathogenicity. EMBO Rep.

[74] Dobrindt U, Agerer F, Michaelis K, Janka A, Buchrieser C, Samuelson M (2003). Analysis of genome plasticity in pathogenic and commensal Escherichia coli isolates by Use of DNA arrays. J Bacteriol.

[75] Gueimonde M, Sánchez BG. de los Reyes-Gavilán C, Margolles A. Antibiotic resistance in probiotic bacteria. Front Microbiol. 2013;4:202. 10.3389/fmicb.2013.00202 PMC3714544 23882264

[76] Cantón R, Novais A, Valverde A, Machado E, Peixe L, Baquero F (2008). Prevalence and spread of extended-spectrum β-lactamase-producing Enterobacteriaceae in Europe. Clin Microbiol Infect.

[77] Livermore DM (1995). beta-lactamases in laboratory and clinical resistance. Clin Microbiol Rev.

[78] Bahramian A, Khoshnood S, Hashemi N, Moradi M, Karimi-Yazdi M, Jalallou N (2021). Identification of metallo-β-lactamases and AmpC production among Escherichia coli strains isolated from hemodialysis patients with urinary tract Infection. Mol Biol Rep.

[79] Fatahi-Bafghi M, Naseri S, Alizehi A (2022). Genome analysis of probiotic bacteria for antibiotic resistance genes. Antonie Van Leeuwenhoek.

[80] Nunziata L, Brasca M, Morandi S, Silvetti T (2022). Antibiotic resistance in wild and commercial non-enterococcal lactic acid Bacteria and bifidobacteria strains of dairy origin: an update. Food Microbiol.

[81] Devirgiliis C, Zinno P, Perozzi G (2013). Update on antibiotic resistance in foodborne Lactobacillus and Lactococcus species. Front Microbiol.

[82] Sirichoat A, Flórez AB, Vázquez L, Buppasiri P, Panya M, Lulitanond V, et al. Antibiotic Resistance-Susceptibility Profiles of Enterococcus faecalis and Streptococcus spp. From the Human Vagina, and Genome Analysis of the Genetic Basis of Intrinsic and Acquired Resistances. Front Microbiol. 2020;11:1438. 10.3389/fmicb.2020.01438 PMC7333779 32695087

[83] Walther C, Rossano A, Thomann A, Perreten V (2008). Antibiotic resistance in Lactococcus species from bovine milk: Presence of a mutated multidrug transporter mdt(A) gene in susceptible Lactococcus garvieae strains. Vet Microbiol.

[84] Ammor MS, Flórez AB, van Hoek AHAM, de los Reyes-Gavilán CG, Aarts HJM, Margolles A (2008). Molecular characterization of intrinsic and acquired antibiotic resistance in lactic acid Bacteria and Bifidobacteria. Microb Physiol.

[85] Leekitcharoenphon P, Johansson MHK, Munk P, Malorny B, Skarżyńska M, Wadepohl K (2021). Genomic evolution of antimicrobial resistance in Escherichia coli. Sci Rep.

[86] De Biase D, Lund PA (2015). The Escherichia coli acid stress response and its significance for pathogenesis..

[87] Miszczycha SD, Thévenot J, Denis S, Callon C, Livrelli V, Alric M (2014). Survival of Escherichia coli O26:H11 exceeds that of Escherichia coli O157:H7 as assessed by simulated human digestion of contaminated raw milk cheeses. Int J Food Microbiol.

[88] Lee Y-K, Salminen S (1995). The coming of age of probiotics. Trends Food Sci Technol.

[89] Han S, Lu Y, Xie J, Fei Y, Zheng G, Wang Z (2021). Probiotic gastrointestinal transit and colonization after oral administration: a long journey. Front Cell Infect Microbiol.

[90] Ageorges V, Monteiro R, Leroy S, Burgess CM, Pizza M, Chaucheyras-durand F (2020). Molecular determinants of surface colonisation in diarrhoeagenic *Escherichia coli* (DEC): from bacterial adhesion to biofilm formation. FEMS Microbiol Rev.

[91] Štaudová B, Micenková L, Bosák J, Hrazdilová K, Slaninková E, Vrba M (2015). Determinants encoding fimbriae type 1 in fecal Escherichia coli are associated with increased frequency of bacteriocinogeny. BMC Microbiol.

[92] Mora M, Bensi G, Capo S, Falugi F, Zingaretti C, Manetti AGO (2005). Group A Streptococcus produce pilus-like structures containing protective antigens and Lancefield T antigens. Proceed National Acad Sci.

[93] Nakata M, Kreikemeyer B (2021). Genetics, structure, and function of Group A Streptococcal Pili. Front Microbiol.

[94] Kimura KR, Nakata M, Sumitomo T, Kreikemeyer B, Podbielski A, Terao Y (2012). Involvement of T6 pili in Biofilm formation by serotype M6 Streptococcus pyogenes. J Bacteriol.

[95] Kleta S, Nordhoff M, Tedin K, Wieler LH, Kolenda R, Oswald S (2014). Role of F1C Fimbriae, Flagella, and secreted bacterial components in the Inhibitory Effect of Probiotic Escherichia coli Nissle 1917 on atypical Enteropathogenic E. Coli Infection. Infect Immun.

[96] Lasaro MA, Salinger N, Zhang J, Wang Y, Zhong Z, Goulian M (2009). F1C fimbriae play an important role in Biofilm formation and intestinal colonization by the *Escherichia coli* Commensal strain Nissle 1917. Appl Environ Microbiol.

[97] Servin AL, Coconnier M-H (2003). Adhesion of probiotic strains to the intestinal mucosa and interaction with pathogens. Best Pract Res Clin Gastroenterol.

[98] Monteagudo-Mera A, Rastall RA, Gibson GR, Charalampopoulos D, Chatzifragkou A (2019). Adhesion mechanisms mediated by probiotics and prebiotics and their potential impact on human health. Appl Microbiol Biotechnol.

[99] Peveri P, Walz A, Dewald B, Baggiolini M (1988). A novel neutrophil-activating factor produced by human mononuclear phagocytes. J Exp Med.

[100] Schröder JM (1989). The monocyte-derived neutrophil activating peptide (NAP/interleukin 8) stimulates human neutrophil arachidonate-5-lipoxygenase, but not the release of cellular arachidonate. J Exp Med.

[101] Sabharwal H, Cichon C, Ölschläger TA, Sonnenborn U, Schmidt MA (2016). Interleukin-8, CXCL1, and MicroRNA miR-146a responses to Probiotic Escherichia coli Nissle 1917 and Enteropathogenic E. Coli in Human intestinal epithelial T84 and monocytic THP-1 cells after apical or basolateral Infection. Infect Immun.

[102] Hafez M, Hayes K, Goldrick M, Warhurst G, Grencis R, Roberts IS (2009). The K5 Capsule of Escherichia coli strain Nissle 1917 is important in mediating interactions with intestinal epithelial cells and chemokine induction. Infect Immun.

[103] Wan MLY, Chen Z, Shah NP, El-Nezami H (2018). Effects of Lactobacillus rhamnosus GG and Escherichia coli Nissle 1917 cell-free supernatants on modulation of mucin and cytokine secretion on human intestinal epithelial HT29-MTX cells. J Food Sci.

[104] Pyrillou K, Burzynski LC, Clarke MCH (2020). Alternative pathways of IL-1 activation, and its role in Health and Disease. Front Immunol.

[105] Vieira MAM, Gomes TAT, Ferreira AJP, Knöbl T, Servin AL, Liévin-Le Moal V (2010). Two atypical Enteropathogenic Escherichia coli strains induce the production of secreted and membrane-bound mucins to benefit their own growth at the apical surface of human mucin-secreting intestinal HT29-MTX cells. Infect Immun.

[106] Wu H, Ye L, Lu X, Xie S, Yang Q, Yu Q (2018). Lactobacillus acidophilus alleviated Salmonella-Induced Goblet cells loss and Colitis by Notch Pathway. Mol Nutr Food Res.

[107] Mack DR, Michail S, Wei S, McDougall L, Hollingsworth MA (1999). Probiotics inhibit enteropathogenic E. Coli adherence in vitro by inducing intestinal mucin gene expression. Am J Physiology-Gastrointestinal Liver Physiol.

[108] Mattar A, Teitelbaum DH, Drongowski R, Yongyi F, Harmon C, Coran A (2002). Probiotics up-regulate MUC-2 mucin gene expression in a Caco-2 cell-culture model. Pediatr Surg Int.

[109] Caballero-Franco C, Keller K, De Simone C, Chadee K (2007). The VSL#3 probiotic formula induces mucin gene expression and secretion in colonic epithelial cells. Am J Physiology-Gastrointestinal Liver Physiol.

[110] Hafez MM (2012). Upregulation of intestinal mucin expression by the Probiotic Bacterium E. Coli Nissle 1917. Probiotics Antimicrob Proteins.

[111] Bishayi B, Bandyopadhyay D, Majhi A, Adhikary R (2015). Effect of exogenous MCP-1 on TLR-2 neutralized murine macrophages and possible mechanisms of CCR-2/TLR-2 and MCP-1 signalling during Staphylococcus aureus Infection. Immunobiology.

[112] Ma TY, Boivin MA, Ye D, Pedram A, Said HM (2005). Mechanism of TNF-α modulation of Caco-2 intestinal epithelial tight junction barrier: role of myosin light-chain kinase protein expression. Am J Physiol Gastrointest Liver Physiol.

[113] Williams CS, DuBois RN (1996). Prostaglandin endoperoxide synthase: why two isoforms?. Am J Physiology-Gastrointestinal Liver Physiol.

[114] Prescott SM, Fitzpatrick FA (2000). Cyclooxygenase-2 and carcinogenesis. Biochimica et Biophysica Acta.

[115] López-Gómez L, Alcorta A, Abalo R (2023). Probiotics and probiotic-like agents against Chemotherapy-Induced Intestinal mucositis: a narrative review. J Pers Med.

[116] Johdi NA, Sukor NF (2020). Colorectal Cancer immunotherapy: options and strategies. Front Immunol.

[117] Gutte R, Deshmukh V (2023). A comprehensive review of the preventive action of natural nutraceutical ingredients in reducing chemotherapy – Induced Side effects. Funct Food Sci.

[118] Dahlgren D, Lennernäs H (2023). Review on the effect of chemotherapy on the intestinal barrier: epithelial permeability, mucus and bacterial translocation. Biomed Pharmacother.

[119] Badgeley A, Anwar H, Modi K, Murphy P, Lakshmikuttyamma A (2021). Effect of probiotics and gut microbiota on anti-cancer drugs: Mechanistic perspectives. Biochim Biophys Acta (BBA) Rev Cancer.

[120] Elad S, Cheng KKF, Lalla RV, Yarom N, Hong C, Logan RM (2020). MASCC/ISOO clinical practice guidelines for the management of mucositis secondary to cancer therapy. Cancer.

[121] Sougiannis AT, VanderVeen BN, Davis JM, Fan D, Murphy EA (2021). Understanding chemotherapy-induced intestinal mucositis and strategies to improve gut resilience. Am J Physiol Gastrointest Liver Physiol.

[122] Sonis ST, Tracey C, Shklar G, Jenson J, Florine D (1990). An animal model for mucositis induced by cancer chemotherapy. Oral Surgery, Oral Medicine, Oral Pathology.

[123] Coelho-Rocha ND, de Jesus LCL, Barroso FAL, da Silva TF, Ferreira E, Gonçalves JE (2023). Evaluation of Probiotic properties of Novel Brazilian lactiplantibacillus plantarum strains. Probiotics Antimicrob Proteins.

[124] Andrade MER, Trindade LM, Leocádio PCL, Leite JIA, dos Reis DC, Cassali GD (2023). Association of Fructo-oligosaccharides and Arginine Improves Severity of Mucositis and modulate the intestinal microbiota. Probiotics Antimicrob Proteins.

[125] Barroso FAL, de Jesus LCL, da Silva TF, Batista VL, Laguna J, Coelho-Rocha ND (2022). Lactobacillus delbrueckii CIDCA 133 ameliorates Chemotherapy-Induced Mucositis by modulating epithelial barrier and TLR2/4/Myd88/NF-κB signaling pathway. Front Microbiol.

[126] Savassi B, Cordeiro BF, Silva SH, Oliveira ER, Belo G, Figueiroa AG (2021). Lyophilized Symbiotic mitigates Mucositis Induced by 5-Fluorouracil. Front Pharmacol.

[127] Américo MF, Freitas ADS, da Silva TF, de Jesus LCL, Barroso FAL, Campos GM (2023). Growth differentiation factor 11 delivered by dairy Lactococcus lactis strains modulates inflammation and prevents mucosal damage in a mice model of intestinal mucositis. Front Microbiol.

[128] Prisciandaro LD, Geier MS, Butler RN, Cummins AG, Howarth GS (2011). Probiotic factors partially improve parameters of 5-fluorouracil-induced intestinal mucositis in rats. Cancer Biol Ther.

[129] Wang H, Jatmiko YD, Bastian SEP, Mashtoub S, Howarth GS (2017). Effects of supernatants from *Escherichia coli* Nissle 1917 and *Faecalibacterium prausnitzii* on intestinal epithelial cells and a rat model of 5-Fluorouracil-Induced Mucositis. Nutr Cancer.

[130] Barbaro MR, Fuschi D, Cremon C, Carapelle M, Dino P, Marcellini MM (2018). *Escherichia coli* Nissle 1917 restores epithelial permeability alterations induced by irritable bowel syndrome mediators. Neurogastroenterology & Motility.

[131] Wang Y, Sun L, Chen S, Guo S, Yue T, Hou Q (2019). The administration of Escherichia coli Nissle 1917 ameliorates irinotecan–induced intestinal barrier dysfunction and gut microbial dysbiosis in mice. Life Sci.

[132] Souza ÉL, Elian SD, Paula LM, Garcia CC, Vieira AT, Teixeira MM (2016). Escherichia coli strain Nissle 1917 ameliorates experimental Colitis by modulating intestinal permeability, the inflammatory response and clinical signs in a faecal transplantation model. J Med Microbiol.

[133] Maioli TU, de Melo Silva B, Dias MN, Paiva NC, Cardoso VN, Fernandes SO (2014). Pretreatment with Saccharomyces boulardii does not prevent the experimental mucositis in Swiss mice. J Negat Results Biomed.

[134] Justino PFC, Melo LFM, Nogueira AF, Morais CM, Mendes WO, Franco AX (2015). Regulatory role of Lactobacillus acidophilus on inflammation and gastric dysmotility in intestinal mucositis induced by 5-fluorouracil in mice. Cancer Chemother Pharmacol.

[135] Yeung CY, Chan WT, Jiang C, Bin, Cheng ML, Liu CY, Chang SW (2015). Amelioration of chemotherapy-induced intestinal mucositis by orally administered probiotics in a mouse model. PLoS ONE.

[136] Tang Y, Wu Y, Huang Z, Dong W, Deng Y, Wang F (2017). Administration of probiotic mixture DM#1 ameliorated 5-fluorouracil–induced intestinal mucositis and dysbiosis in rats. Nutrition.

[137] De Jesus LCL, Drumond MM, de Carvalho A, Santos SS, Martins FS, Ferreira Ê (2019). Protective effect of Lactobacillus delbrueckii subsp. Lactis CIDCA 133 in a model of 5 Fluorouracil-Induced intestinal mucositis. J Funct Foods.

[138] Cordeiro BF, Oliveira ER, Da Silva SH, Savassi BM, Acurcio LB, Lemos L (2018). Whey protein isolate-supplemented Beverage, fermented by Lactobacillus casei BL23 and Propionibacterium freudenreichii 138, in the Prevention of Mucositis in mice. Front Microbiol.

[139] Frank DN, St. Amand AL, Feldman RA, Boedeker EC, Harpaz N, Pace NR (2007). Molecular-phylogenetic characterization of microbial community imbalances in human inflammatory bowel diseases. Proceed National Acad Sci.

[140] Ma X, Lu X, Zhang W, Yang L, Wang D, Xu J (2022). Gut microbiota in the early stage of Crohn’s Disease has unique characteristics. Gut Pathog.

[141] Algieri F, Garrido-Mesa J, Vezza T, Rodríguez-Sojo MJ, Rodríguez-Cabezas ME, Olivares M (2021). Intestinal anti-inflammatory effects of probiotics in DNBS-colitis via modulation of gut microbiota and microRNAs. Eur J Nutr.

[142] Cui Y, Zhang L, Wang X, Yi Y, Shan Y, Liu B (2022). Roles of intestinal Parabacteroides in human health and Diseases. FEMS Microbiol Lett.

[143] Mukherjee A, Lordan C, Ross RP, Cotter PD (2020). Gut microbes from the phylogenetically diverse genus *Eubacterium* and their various contributions to gut health. Gut Microbes.

[144] Kasahara K, Krautkramer KA, Org E, Romano KA, Kerby RL, Vivas EI (2018). Interactions between Roseburia intestinalis and diet modulate atherogenesis in a murine model. Nat Microbiol.

[145] Chang S-C, Shen M-H, Liu C-Y, Pu C-M, Hu J-M, Huang C-J. A gut butyrate-producing bacterium Butyricicoccus pullicaecorum regulates short-chain fatty acid transporter and receptor to reduce the progression of 1,2-dimethylhydrazine-associated colorectal cancer. Oncol Lett. 2020;20:327. 10.3892/ol.2020.12190 PMC7577080 33101496

[146] Ćesić D, Lugović Mihić L, Ozretić P, Lojkić I, Buljan M, Šitum M (2023). Association of Gut Lachnospiraceae and Chronic spontaneous urticaria. Life.

[147] Kang G-U, Park S, Jung Y, Jee JJ, Kim M-S, Lee S (2022). Exploration of potential gut microbiota-derived biomarkers to predict the success of fecal microbiota transplantation in Ulcerative Colitis: a prospective cohort in Korea. Gut Liver.

[148] Gao Z, Yin J, Zhang J, Ward RE, Martin RJ, Lefevre M (2009). Butyrate improves insulin sensitivity and increases Energy Expenditure in mice. Diabetes.

[149] Shang Q, Song G, Zhang M, Shi J, Xu C, Hao J (2017). Dietary fucoidan improves metabolic syndrome in association with increased Akkermansia population in the gut microbiota of high-fat diet-fed mice. J Funct Foods.

[150] Hao W, Zhu H, Chen J, Kwek E, He Z, Liu J (2020). Wild melon seed oil reduces plasma cholesterol and modulates gut microbiota in Hypercholesterolemic Hamsters. J Agric Food Chem.

[151] Harrison CA, Laubitz D, Ohland CL, Midura-Kiela MT, Patil K, Besselsen DG (2018). Microbial dysbiosis associated with impaired intestinal Na+/H + exchange accelerates and exacerbates Colitis in ex-germ free mice. Mucosal Immunol.

[152] Beller A, Kruglov A, Durek P, von Goetze V, Werner K, Heinz GA (2020). Specific microbiota enhances intestinal IgA levels by inducing TGF-β in T follicular helper cells of Peyer’s patches in mice. Eur J Immunol.

[153] Antipov D, Korobeynikov A, McLean JS, Pevzner PA (2016). hybridSPAdes: an algorithm for hybrid assembly of short and long reads. Bioinformatics.

[154] Robertson J, Nash JHE (2018). MOB-suite: software tools for clustering, reconstruction and typing of plasmids from draft assemblies. Microb Genom.

[155] Guizelini D, Raittz RT, Cruz LM, Souza EM, Steffens MBR, Pedrosa FO (2016). GFinisher: a new strategy to refine and finish bacterial genome assemblies. Sci Rep.

[156] Hernandez D, Tewhey R, Veyrieras JB, Farinelli L, Østerås M, François P (2014). De novo finished 2.8 mbp Staphylococcus aureus genome assembly from 100 bp short and long range paired-end reads. Bioinformatics.

[157] Li W, O’Neill KR, Haft DH, DiCuccio M, Chetvernin V, Badretdin A (2021). RefSeq: expanding the Prokaryotic Genome Annotation Pipeline reach with protein family model curation. Nucleic Acids Res.

[158] Haft DH, DiCuccio M, Badretdin A, Brover V, Chetvernin V, O’Neill K (2018). RefSeq: an update on prokaryotic genome annotation and curation. Nucleic Acids Res.

[159] Tatusova T, DiCuccio M, Badretdin A, Chetvernin V, Nawrocki EP, Zaslavsky L (2016). NCBI prokaryotic genome annotation pipeline. Nucleic Acids Res.

[160] Cantalapiedra CP, Hernández-Plaza A, Letunic I, Bork P, Huerta-Cepas J (2021). eggNOG-mapper v2: functional annotation, Orthology assignments, and Domain Prediction at the Metagenomic Scale. Mol Biol Evol.

[161] Huerta-Cepas J, Szklarczyk D, Heller D, Hernández-Plaza A, Forslund SK, Cook H (2019). eggNOG 5.0: a hierarchical, functionally and phylogenetically annotated orthology resource based on 5090 organisms and 2502 viruses. Nucleic Acids Res.

[162] Emms DM, Kelly S (2019). OrthoFinder: phylogenetic orthology inference for comparative genomics. Genome Biol.

[163] Davis JJ, Gerdes S, Olsen GJ, Olson R, Pusch GD, Shukla M (2016). PATtyFams: protein families for the Microbial genomes in the PATRIC Database. Front Microbiol.

[164] Edgar RC (2004). MUSCLE: multiple sequence alignment with high accuracy and high throughput. Nucleic Acids Res.

[165] Stamatakis A (2014). RAxML version 8: a tool for phylogenetic analysis and post-analysis of large phylogenies. Bioinformatics.

[166] Stamatakis A, Hoover P, Rougemont J (2008). A Rapid bootstrap algorithm for the RAxML web servers. Syst Biol.

[167] Soares SC, Geyik H, Ramos RTJ, de Sá PHCG, Barbosa EGV, Baumbach J (2016). GIPSy: genomic island prediction software. J Biotechnol.

[168] Arndt D, Grant JR, Marcu A, Sajed T, Pon A, Liang Y (2016). PHASTER: a better, faster version of the PHAST phage search tool. Nucleic Acids Res.

[169] Zhou Y, Liang Y, Lynch KH, Dennis JJ, Wishart DS (2011). PHAST: a fast Phage Search Tool. Nucleic Acids Res.

[170] Alikhan N-F, Petty NK, Ben Zakour NL, Beatson SA (2011). BLAST Ring Image Generator (BRIG): simple prokaryote genome comparisons. BMC Genomics.

[171] van Heel AJ, de Jong A, Song C, Viel JH, Kok J, Kuipers OP (2018). BAGEL4: a user-friendly web server to thoroughly mine RiPPs and bacteriocins. Nucleic Acids Res.

[172] Brodkorb A, Egger L, Alminger M, Alvito P, Assunção R, Ballance S (2019). INFOGEST static in vitro simulation of gastrointestinal food digestion. Nat Protoc.

[173] da Luz BSR, de Rezende Rodovalho V, Nicolas A, Chabelskaya S, Jardin J, Briard-Bion V (2022). Impact of environmental conditions on the protein content of Staphylococcus aureus and its derived extracellular vesicles. Microorganisms.

[174] Tarnaud F, Gaucher F, do Carmo FLR, Illikoud N, Jardin J, Briard-Bion V (2020). Differential Adaptation of Propionibacterium freudenreichii CIRM-BIA129 to Cow’s Milk Versus Soymilk Environments Modulates Its Stress Tolerance and Proteome. Front Microbiol.

[175] Langella O, Valot B, Balliau T, Blein-Nicolas M, Bonhomme L, Zivy M (2017). X!TandemPipeline: A Tool to manage sequence redundancy for protein inference and Phosphosite Identification. J Proteome Res.

[176] Rodovalho V, de R BSR, Rabah H, do Carmo FLR, Folador EL, Nicolas A (2020). Extracellular vesicles produced by the Probiotic Propionibacterium freudenreichii CIRM-BIA 129 mitigate inflammation by modulating the NF-κB pathway. Front Microbiol.

[177] Ishihama Y, Oda Y, Tabata T, Sato T, Nagasu T, Rappsilber J (2005). Exponentially modified protein abundance index (emPAI) for estimation of absolute protein amount in proteomics by the number of sequenced peptides per protein. Mol Cell Proteomics.

[178] Howarth GS, Francis GL, Cool JC, Xu X, Byard RW, Read LC (1996). Milk Growth Factors Enriched from Cheese Whey Ameliorate Intestinal Damage by Methotrexate When Administered Orally to Rats. J Nutr.

[179] Bolger AM, Lohse M, Usadel B (2014). Trimmomatic: a flexible trimmer for Illumina sequence data. Bioinformatics.

[180] Schloss PD, Westcott SL, Ryabin T, Hall JR, Hartmann M, Hollister EB (2009). Introducing mothur: Open-Source, Platform-Independent, community-supported Software for describing and comparing Microbial communities. Appl Environ Microbiol.

[181] Bolyen E, Rideout JR, Dillon MR, Bokulich NA, Abnet CC, Al-Ghalith GA (2019). Reproducible, interactive, scalable and extensible microbiome data science using QIIME 2. Nat Biotechnol.

[182] Quast C, Pruesse E, Yilmaz P, Gerken J, Schweer T, Yarza P (2012). The SILVA ribosomal RNA gene database project: improved data processing and web-based tools. Nucleic Acids Res.

