# Supplementary material for: Comprehensive probiogenomics analysis of the commensal Escherichia coli CEC15 as a potential probiotic strain
Source: BMC Microbiol. 2023 Nov 27;23:364. doi: 10.1186/s12866-023-03112-4 (PMC10680302; doi:10.1186/s12866-023-03112-4)
Supplement: Supplementary file 8 — Additional file 8: Supplementary Figure S2. Schematic circular representation of EcN genomic features. [file 12866_2023_3112_MOESM8_ESM.docx]

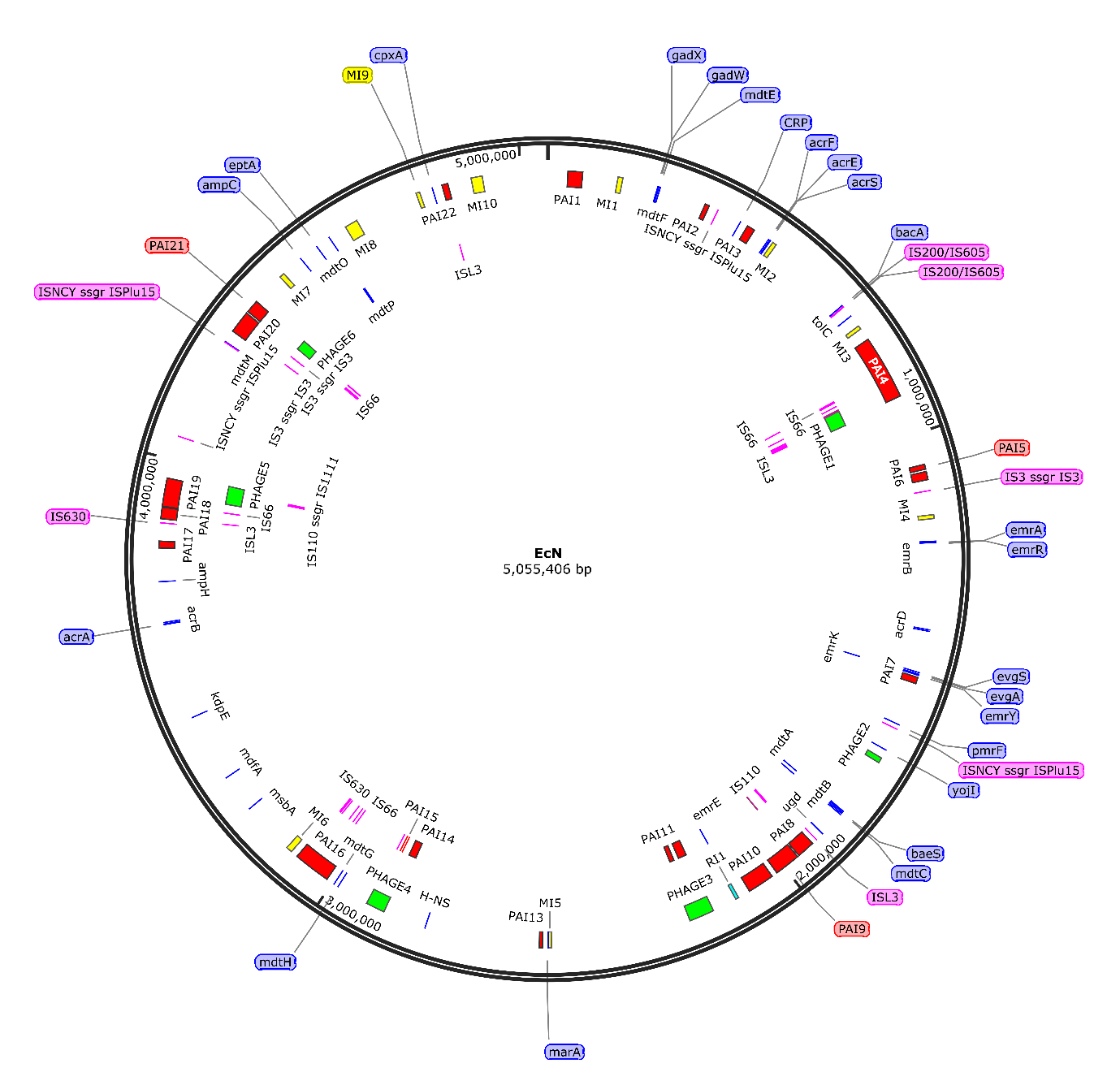


**Supplementary Figure S2. Schematic circular representation of EcN genomic features.** Pathogenicity Island (PAI) (red), Metabolic Island (MI) (yellow), Resistance Island (RI) (cyan) Prophage regions (PHAGE) (green), Insertion elements (IS) (pink), and antibiotic resistance-related genes (blue) are represented in their respective position in the genome. Constructed using SnapGene Viewer software (from Insightful Science; available at snapgene.com).
