# Supplementary material for: Comprehensive probiogenomics analysis of the commensal Escherichia coli CEC15 as a potential probiotic strain
Source: BMC Microbiol. 2023 Nov 27;23:364. doi: 10.1186/s12866-023-03112-4 (PMC10680302; doi:10.1186/s12866-023-03112-4)
Supplement: Supplementary file 13 — Additional file 13: Supplementary figure S3. Pre- and post-shearing protein profile of CEC15 and EcN strains. [file 12866_2023_3112_MOESM13_ESM.docx]

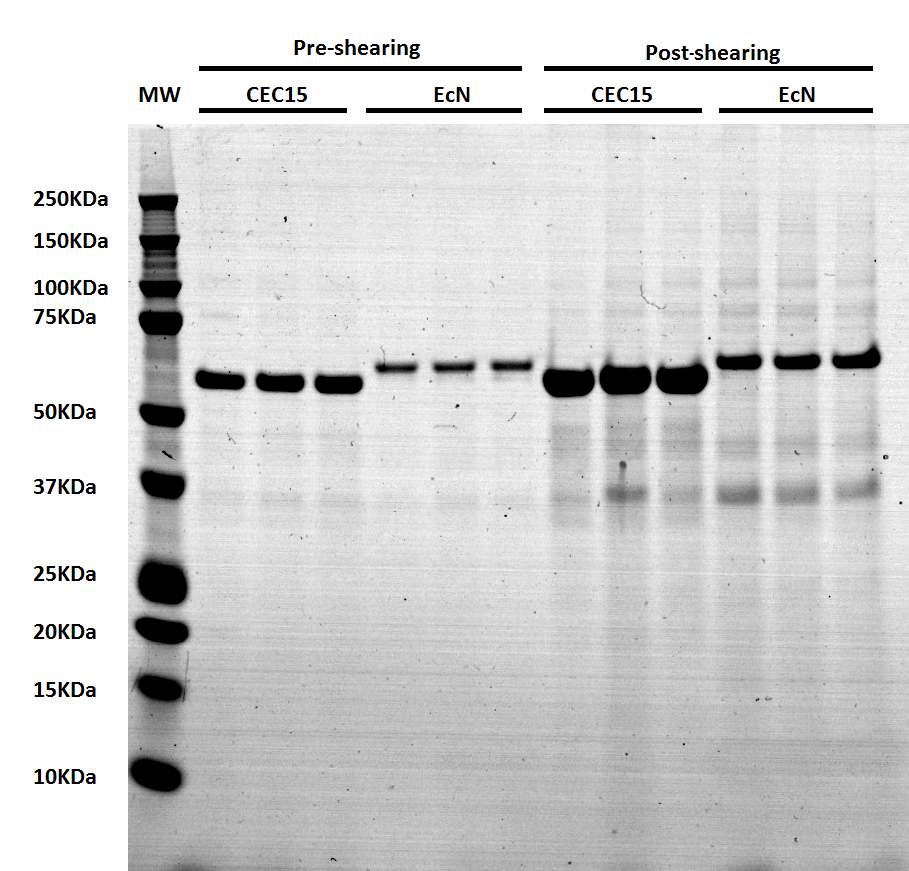


**Supplementary figure S3. Pre- and post-shearing protein profile of CEC15 and EcN strains.** Strains were grown overnight at 37 °C, without agitation, centrifuged, and resuspended in PBS at 1:100 the initial volume. One aliquot was collected for the pre-shearing and the samples were blended for 5 min at maximum speed with an waring blender (Waring 2-Speed Blender, Vican, USA). Aliquots pre- and post-shearing were centrifuged and the supernatant resolved in SDS-PAGE gradient gel (4-12%).
