# Supplementary material for: Comprehensive probiogenomics analysis of the commensal Escherichia coli CEC15 as a potential probiotic strain
Source: BMC Microbiol. 2023 Nov 27;23:364. doi: 10.1186/s12866-023-03112-4 (PMC10680302; doi:10.1186/s12866-023-03112-4)
Supplement: Supplementary file 15 — Additional file 15: Supplementary figure S4. Bacteriocins-encoding genes present in the genome of CEC15 and EcN strains. [file 12866_2023_3112_MOESM15_ESM.docx]

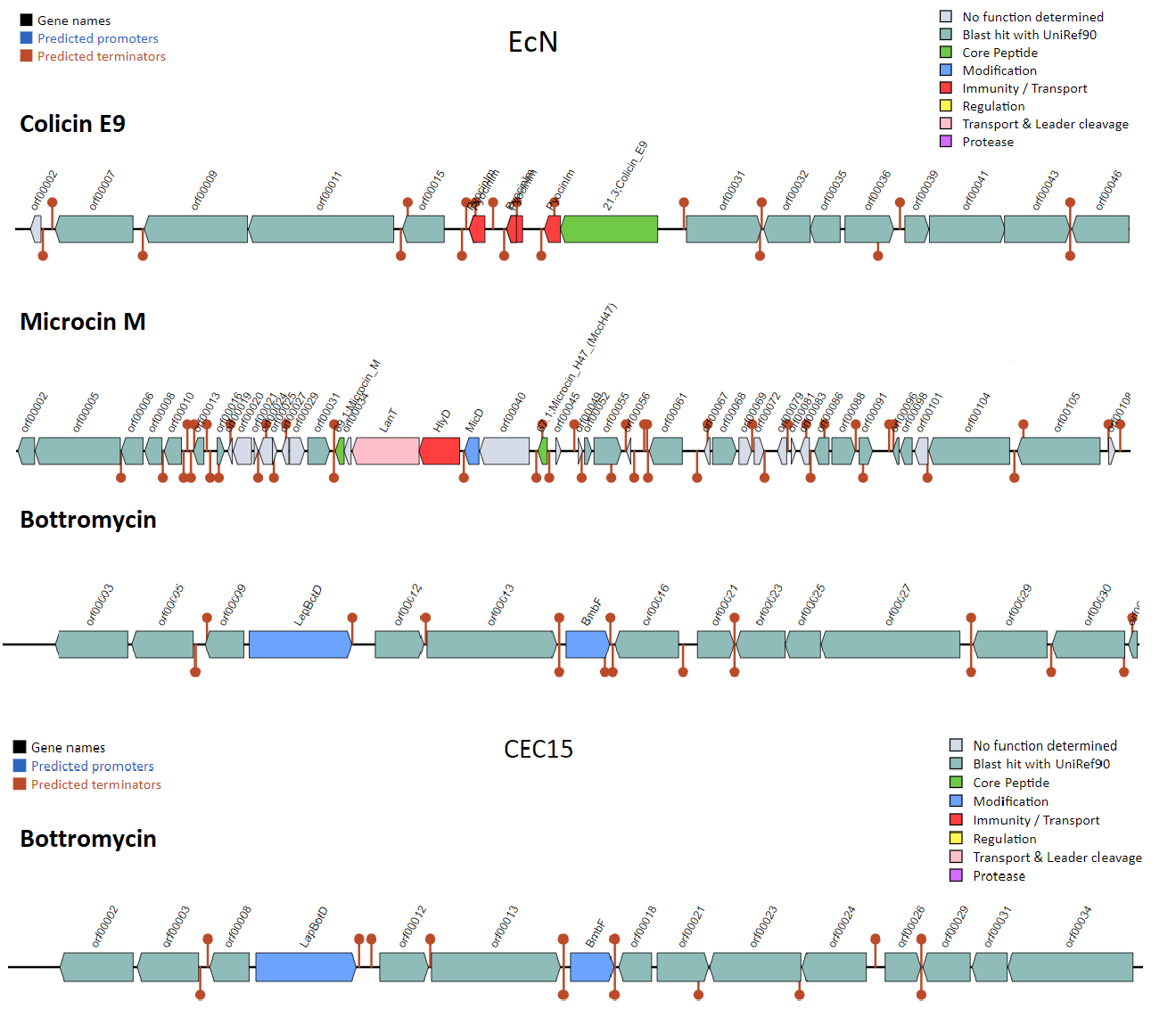


**Supplementary figure S4. Bacteriocins-encoding genes present in the genome of CEC15 and EcN strains**. The identification of bacteriocins-encoding genes were performed by matching the genome fasta files with the BAGEL4 database (<http://bagel4.molgenrug.nl/>). The image shows the cluster of genes related to each bacteriocin in each strain with the core sequence being highlighted in green. Tree bacteriocin-encoding genes were found on EcN strain (Colicin E9, Microcin M, and Bottromycin) while only one (Bottromycin) was found on CEC15.
