# Supplementary material for: Comprehensive probiogenomics analysis of the commensal Escherichia coli CEC15 as a potential probiotic strain
Source: BMC Microbiol. 2023 Nov 27;23:364. doi: 10.1186/s12866-023-03112-4 (PMC10680302; doi:10.1186/s12866-023-03112-4)
Supplement: Supplementary file 16 — Additional file 16: Supplementary figure S5. Relative gene expression of Caco-2 cells is unaltered by treated with either CEC15 or EcN strains [file 12866_2023_3112_MOESM16_ESM.docx]

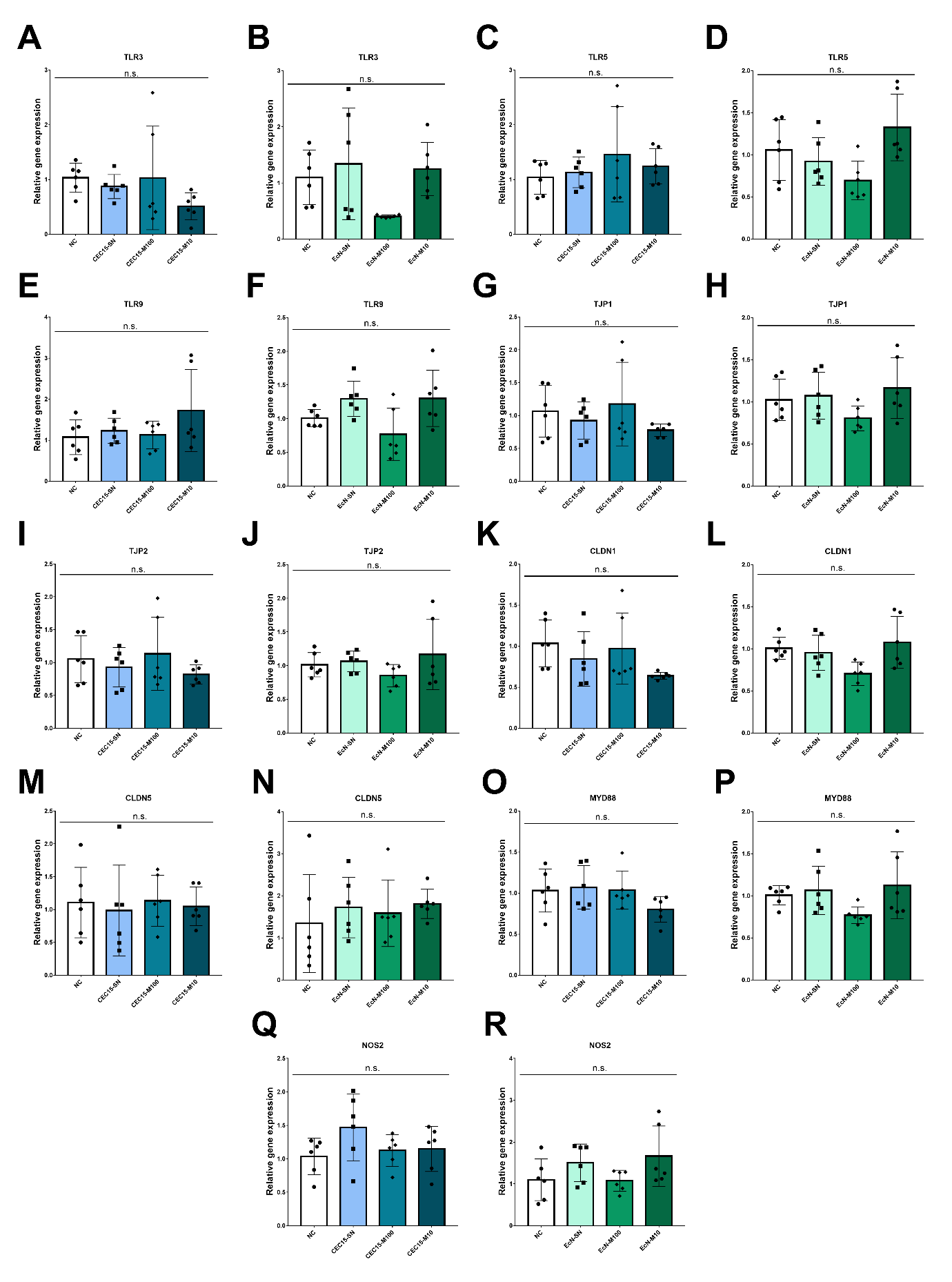


**Supplementary figure S5. Relative gene expression of Caco-2 cells is unaltered by treated with either CEC15 or EcN strains.** Gene expression were normalized by the reference genes GAPDH, B2M, and HPRT1. Statistical analysis was performed by one-way ANOVA with the post-test of Tukey using the GraphPad Prism 7.0. n.s. indicated no significative difference between groups. NC: negative control; CEC15-SN: CEC15 supernatant; CEC15-M100: CEC15 treatment at MOI 100; CEC15-M10: CEC15 treatment at MOI 10; EcN-SN: EcN supernatant; EcN-M100: EcN treatment at MOI 100; EcN-M10: EcN15 treatment at MOI 10.
